# Origin of Excellent Charge Storage Properties of Defective Tin Disulphide in Magnesium/Lithium-Ion Hybrid Batteries

**DOI:** 10.1007/s40820-022-00914-5

**Published:** 2022-08-24

**Authors:** Xin Fan, Mike Tebyetekerwa, Yilan Wu, Rohit Ranganathan Gaddam, Xiu Song Zhao

**Affiliations:** 1 School of Chemical Engineering, The University of Queensland, St Lucia, Brisbane, QLD 4072 Australia; 2 School of Material Science and Technology, North University of China, Taiyuan, 030051 Shanxi People’s Republic of China; 3 Dow Centre for Sustainable Engineering Innovation, School of Chemical Engineering, The University of Queensland, St Lucia, Brisbane, QLD 4072 Australia; 4 Department of Chemical Engineering, Indian Institute of Science Education and Research, Bhopal, India

**Keywords:** Defects, Tin disulphide, Magnesium/lithium-ion hybrid batteries, 2D materials

## Abstract

Lithium-ion batteries (LIBs) are excellent electrochemical energy sources, albeit with existing challenges, including high costs and safety concerns. Magnesium-ion batteries (MIBs) are one of the potential alternatives. However, the performance of MIBs is poor due to their sluggish solid-state Mg^2+^ diffusion kinetics and severe electrode polarizability. Rechargeable magnesium-ion/lithium-ion (Mg^2+^/Li^+^) hybrid batteries (MLHBs) with Mg^2+^ and Li^+^ as the charge carriers create a synergy between LIBs and MIBs with significantly improved charge transport kinetics and reliable safety features. However, MLHBs are yet to reach a reasonable electrochemical performance as expected. This work reports a composite electrode material with highly defective two-dimensional (2D) tin sulphide nanosheets (SnS_*x*_) encapsulated in three-dimensional (3D) holey graphene foams (HGF) (SnS_*x*_/HGF), which exhibits a specific capacity as high as 600 mAh g^−1^ at 50 mA g^−1^ and a compelling specific energy density of ~ 330 Wh kg^−1^. The excellent electrochemical performance surpasses previously reported hybrid battery systems based on intercalation-type cathode materials under comparable conditions. The role played by the defects in the SnS_*x*_/HGF composite is studied to understand the origin of the observed excellent electrochemical performance. It is found that it is closely related to the defect structure in SnS_*x*,_ which offers percolation pathways for efficient ion transport and increased internal surface area assessable to the charge carriers. The defective sites also absorb structural stress caused by Mg^2+^ and Li^+^ insertion. This work is an important step towards realizing high-capacity cathode materials with fast charge transport kinetics for hybrid batteries.

**Supplementary Information:**

The online version contains supplementary material available at 10.1007/s40820-022-00914-5.

## Highlights

- Defects in SnS_x_ play an important role in enhancing charge storage capacity and transport kinetics in magnesium/lithium-ion hybrid batteries.
- Defective SnS_x_ is a promising conversion-type cathode for magnesium/lithium-ion hybrid batteries.
- The co-insertion of Mg^2+^/Li^+^ enhances hybrid battery performance.

**Supplementary Information:**

The online version contains supplementary material available at 10.1007/s40820-022-00914-5.

## Graphical abstract

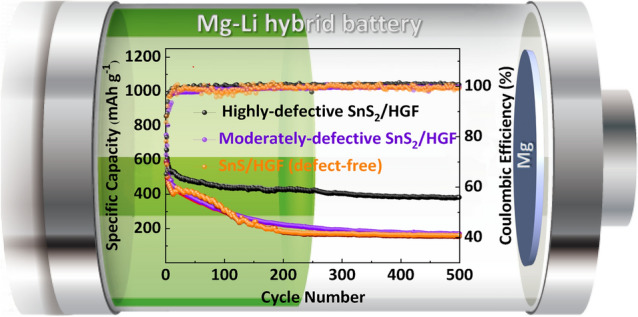

## Introduction

Lithium-ion batteries (LIBs) are currently the key to realizing a fossil-fuel-free economy. Their global demand between 2020 and 2030 is predicted to increase 11-fold to a tune of over two terawatt-hours in the year 2030 [1]. The key driving factors are the anticipated transition to a green circular and renewable economy, increased portable electronics, and the rising popularity of electric vehicles and locomotives, which predominantly rely on LIBs for power [2]. However, LIBs face several grand challenges hindering their further market thrust, such as safety, limited lithium resources and associated raising cost [3, 4]. These challenges have triggered the investigation of novel battery systems as potential replacements for LIBs [5]. One of these alternatives is magnesium-ion batteries (MIBs) featuring magnesium which is inexpensive, safe to handle, environmentally friendly making them an excellent choice in rechargeable batteries, with a possibility to deliver a considerably higher energy density [6]. Compared with LIBs, the use of bivalent Mg^2+^ as the charge carrier can potentially enable a higher volumetric energy density (3832 and 2062 mAh cm^−3^ for Mg and Li, respectively [7]. In addition, metal magnesium (Mg) as an anode is less prone to dendritic growth than Li [5, 8–10]. Hence, MIBs provide fewer safety concerns than LIBs and can improve energy density by up to about 4–5 times from only the anode standpoint. However, one of the key challenges facing the MIB technology is the sluggish solid-state diffusion kinetics of Mg^2+^ in host materials due to its high polarization (the ionic charge density of Mg^2+^ is 120 vs. 52 C mm^−3^ of Li^+^) [11]. Hence, this could lead to low Mg^2+^ intercalation level and large voltage hysteresis for common cathode materials.

Rechargeable Mg^2+^/Li^+^ hybrid batteries (MLHBs) consisting of Mg metal anode, Li^+^ intercalation cathode, and dual-salt electrolyte with both solvated Mg^2+^ and Li^+^ cations are excellent synergistic options interlinking LIBs and MIBs with significantly improved charge transport kinetics [12]. This is because, with their cell design, the Li^+^ intercalation/deintercalation occurs at the cathode with a fast kinetic rate, whereas the advantages of using metal Mg as anode are maintained, because the anode side is dominated by the Mg^2+^/Mg deposition/dissolution [13, 14]. In such hybrid batteries, since the dual-ion electrolyte is the only reservoir to supply Li^+^ to the cathode, the amount of electrolyte and the concentration of Li^+^ would directly affect their reaction behaviours. The addition of Li salts brings good reaction kinetics to the cathodes and increases the electrochemical activity of Mg deposition/dissolution [15, 16]. The reason for the former is evident that the dominant reaction at the cathode is governed by Li^+^ insertion, which bypasses the sluggish transportation of Mg^2+^. The latter is attributed to the higher ionic conductivities, significantly reduced interfacial resistance, and improved interfacial compatibility between the electrolyte and Mg metal anode [14]. In addition, our previous work [13] and many other works [17, 18] reported that the prevailing Li^+^ insertions can reduce the migration barrier and activation energy of the subsequent Mg^2+^ insertion, resulting in remarkable electrochemical performances. However, the addition of Li salts would also narrow down the voltage window of the complex electrolyte. Moreover, the solubility of Li salts limits the concentration of Li^+^ within ethereal solvents, which requires a large amount of electrolyte solvent with enough Li^+^ (normally 0.5 M as shown in Table S1) to ensure high capacity. This issue drags the anticipated significant improvement of the cell performance [19, 20]. Many strategies exist to work around this problem, with electrolyte and electrode engineering being more practical [14].

For the case of electrode engineering, to improve cell performance, there is a need to develop new electrode materials enabling efficient multi-electron transfer within the proper voltage window. Several aspects must be considered when choosing a cathode material for high-performance MLHBs. First, the chosen cathode must be compatible with the complex electrolyte. For example, it should have redox potential within the complex electrolyte’s electrochemical stability window and must remain chemically inert to electrolyte components to avoid parasitic reactions. Second, considering the limited quantity of Li^+^ in MLHB electrolytes, the ideal cathode material should be able to accommodate both Mg^2+^ and Li^+^. In this case (as illustrated in Scheme S1), both Mg^2+^ and Li^+^ insertion and de-insertion occur at the cathode. The advantages of using Mg anode are retained as Mg is electrodeposited preferentially at the anode with a higher redox potential of Mg^2+^/Mg (− 2.37 V vs. SHE) than Li^+^/Li (− 3.04 V vs. SHE) [14, 21]. Compared with the asymmetric use of Mg^2+^ and Li^+^ on cathode and anode separately, the use of Mg^2+^/Li^+^ co-intercalation-type cathodes can improve the energy density of the whole battery by increasing the utilization rate of Mg^2+^ (both Mg^2+^ and Li^+^ ions can participate in cathode side reactions) and reducing the amount of electrolyte. Therefore, strategies to minimize diffusion barriers of Mg^2+^ can be applied in the search and modification of high-performance Mg hybrid batteries cathodes. This can involve size tailoring strategy, doping heteroatoms into the crystal lattice, changing materials structures and morphologies, creating material heterostructures, synthesizing hybrids materials, and many other approaches [20, 22].

The existing inorganic cathode materials are usually considered the starting materials as they are already promising. Considering the relatively narrow voltage window of MLHB electrolytes (< 3.0 V vs. Mg^2+^/Mg), it is important to consider cathode materials that can offer high theoretical capacities for improved energy density. Compared to the most studied intercalation-type cathode materials, the conversion-type materials can provide higher theoretical capacities with the multi-electron transfer reactions [23, 24]. One example of such materials is tin sulphide (SnS_*x*_, where *x* = 1 or 2), which can store both small ions (such as Li^+^ [25, 26]) and big ions (such as Na^+^ [27, 28]) towards high-capacity battery electrodes. However, the cycle stability of SnS_*x*_ is poor due to the common problems associated with conversion reactions, like large volumetric expansion, large voltage hysteresis, and low conversion efficiency, which are hardly avoidable [24]. Besides, due to the fundamental diffusion limitation of Mg^2+^ in solid state, achieving high ion mobility within the solid electrodes is a prerequisite for realizing a proper conversion-type cathode for Mg^2+^ storage. Nevertheless, with the known advantages and limitations, appropriate approach(es) towards a new and better electrode material based on SnS_*x*_ enabling efficient multi-electron transfer can be realized.

In this work, these crucial challenges were well addressed via synergistic modulations and improvement of both structure and conductivity of SnS_*x*_. We rationally design and study SnS_*x*_*-*based materials to understand and reveal the role played by defects and their structures to their electrochemical performance as advanced cathode materials for MLHBs. Briefly, first, we prepare two-dimensional (2D) highly defective, moderately defective, and defect-free tin (iv) disulphide (SnS_2_) in the presence of three-dimensional (3D) holey graphene foams (HGF) to encapsulate the 2D SnS_*x*_ sheets towards improved electron conductivity. From these, with systematic experimental and characterization studies, we reveal that defects are key to improving the electrochemical performance of 2D SnS_2_ sheets. This is because they act as active sites for charge storage and as channels for ion transport. In addition, vacancy defects can absorb stress caused by structural deformations during charging and discharging. In brief, the highly defective SnS_2_/HGF demonstrate a high specific capacity of 600 mAh g^−1^ at 50 mA g^−1^ and 205 mAh g^−1^ at 1 A g^−1^. And, even after 500 cycles at 800 mA g^−1^, the specific capacity remains 160 mAh g^−1^. Through these findings, to the best of our knowledge, these SnS_*x*_/HGF cathodes in MLHB are superior to other insertion cathodes previously reported in hybrid batteries under comparable rates. Therefore, this work realizes the aim of using high-capacity materials to accommodate fast kinetics.

## Experimental Section

### Material Preparation and Characterization

Natural graphite powder was oxidized to graphene oxide (GO) using the modified Hummers’ method. The SnS_2_/HGF composite was prepared from a one-step hydrothermal approach as described below. Typically, tin (IV) chloride (0.79 g) was dissolved in 40 mL deionized (DI) water under vigorous stirring. Afterwards, 5 mL of 2 mg mL^−1^ graphene oxides suspension containing 30 μL H_2_O_2_ was added to the as-prepared Tin precursor aqueous solution followed by 1 h sonication treatment. Then, thiourea (1.38 g for highly defective SnS_2_/HGF and 0.47 g for moderately defective SnS_2_/HGF) was sequentially added, and the mixture was transferred to a 50 mL Teflon-lined stainless steel autoclave and heated at 160 °C for 24 h. After cooling to ambient temperature, the solids of black in colour was collected by centrifugation, washed with DI water and ethanol, repeatedly, and then freeze-dried for 48 h. A defect-free sample, denoted as SnS/HGF, was obtained by further annealing the moderately defective SnS_2_/HGF at 600 °C for 2 h in a tube furnace in nitrogen flow.

The morphologies of as-prepared samples were examined by using a field-emission scanning electron microscope (FESEM) JEOL 7001 at 10 kV. Transmission electron microscopy (TEM) and high-resolution TEM (HRTEM) measurements were conducted on a JEOL-JEM-2100F microscope equipped with an energy dispersive X-ray (EDX). X-ray diffraction (XRD) patterns were collected on a Bruker D8 Advance X-ray diffractometer with Ni-filtered Cu Kα radiation at a scan rate of 2 degrees min^−1^. X-ray photoelectron spectroscopy (XPS) spectra were acquired on a Kratos Axis ULTRA X-ray photoelectron spectrometer, with the C 1 s binding energy of 284.8 eV as the reference for calibrating the binding energies of other elements. Raman spectra were collected on a Renishaw Raman spectrometer with a 514 nm laser source.

### Electrochemical Measurements

The MIBs and MLHBs were assembled in a 2032-type coin cell with the SnS_*x*_-HGF (*x* = 1 or 2) composite as the cathode, magnesium ribbon (Sigma-Aldrich, 13,103) as the anode, and Celgard 3501 as the separator. All electrodes and separators were dried at 80 °C in a vacuum oven overnight before use. A 0.25 M all phenyl complex (APC) electrolyte for MIB was synthesized in a glove box filled with high purity argon. A dual-salt electrolyte containing both APC and lithium chloride was used to fabricate the hybrid battery cell. Note that the presence of Li^+^ in the electrolyte was reported to favour the reactivity of Mg [29, 30]. Besides, the Cl^−^ in the solution also plays an important role on the improvement of electrochemical performance of batteries [31]. The cyclic voltammetry measurements were carried out on a CHI-600D electrochemical workstation at a scan rate of 0.2 mV s^−1^ in the voltage range between 0.01 and 2 V. Electrochemical impedance spectroscopy measurements were also performed on the CHI 660D electrochemical workstation in the frequency range between 100 and 10 mHz. The cycle performance and charge/discharge profiles were measured on a Neware battery tester CT3008.

## Results and Discussion

### Structural Analysis

**Scheme 1:**
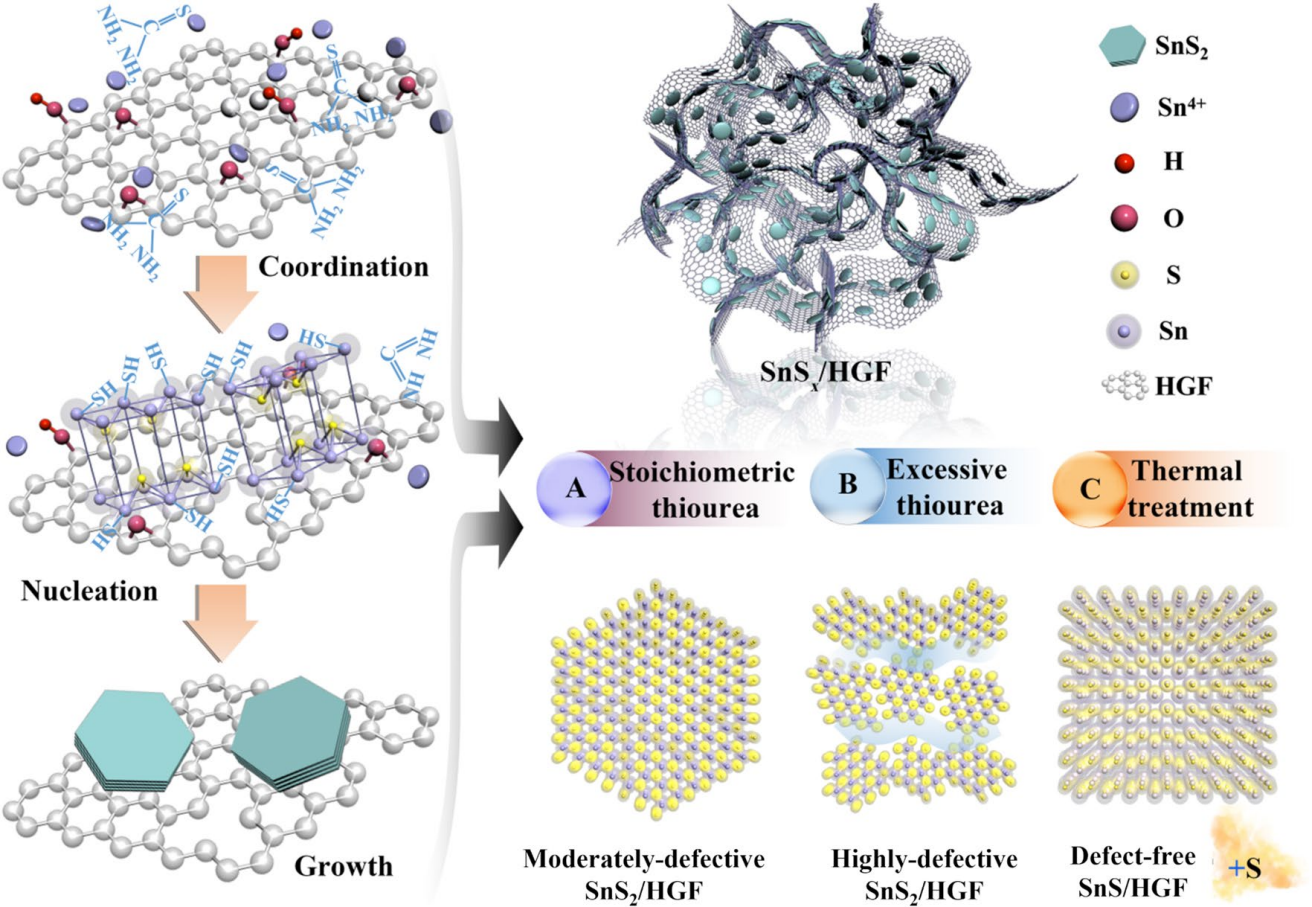
Illustration of the preparation of the samples studied in this work

To prepare highly conductive SnS_*x*_ composites, 2D SnS_*x*_ sheets are first introduced to 3D HGF in which they are firmly encapsulated. Here, the highly defective SnS_2_ sample is synthesized in the presence of an excessive amount of thiourea as the sulphurizing agent. The surface functional groups on GO play an important role in anchoring the Sn^4+^ and thiourea onto its surface. The S atom in thiourea coordinates with the Sn^4+^ to yield a Sn-thiourea complex [32]. In all samples, the nucleation and particle growth of SnS_2_ happens at 160 °C during the hydrothermal process. The presence of excessive thiourea in producing highly defective SnS_2_/HGF provides sufficient monomer to facilitate the nucleation process [33] and tends to restrict the formation of large crystals [34]. Thus enabling the construction of the defect-rich structure with quasi-periodic configuration. For comparison purposes, the moderately defective SnS_2_/HGF sample is also synthesized under the same conditions except with a stoichiometric amount of thiourea. Thermal treatment of the moderately defective SnS_2_/HGF at 600 °C in nitrogen atmosphere is further carried out to obtain defect-free SnS_*x*_/HGF sample (SnS/HGF). During the thermal treatment process, defective sites tend to get restored with the process realizing excessive sulphur depletion according to the following equation: SnS_2_(s) → SnS(s) + 1/*x* S_*x*_(g) [27]. The illustrations in Scheme 1 summarizes the preparation procedures above.

**Fig. 1:**
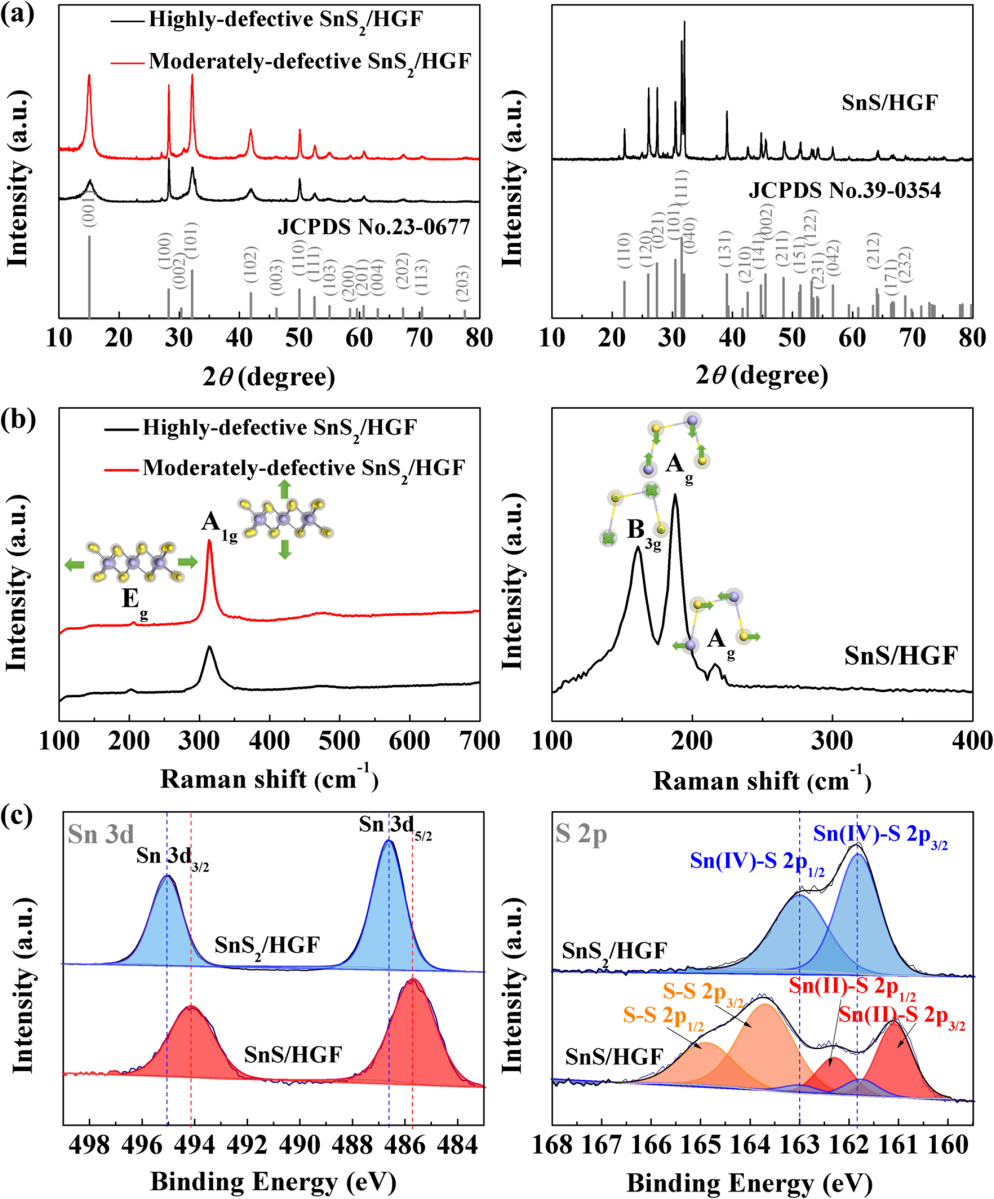
**a** XRD patterns of highly defective SnS_2_/HGF and moderately defective SnS_2_/HGF (the left figure) and defect-free SnS/HGF (the right figure); **b** Raman spectra of highly defective SnS_2_/HGF and moderately defective SnS_2_/HGF (the left figure) and SnS/HGF (the right figure); **c** XPS spectra of Sn 3*d* (the left figure) and S 2*p* (the right figure) of highly defective SnS_2_/HGF and defect-free SnS/HGF

To confirm the synthesis and design of associated materials, XRD patterns, Raman spectroscopy, XPS, FESEM, and HRTEM characterizations are carried out in the first part of the study. Starting with XRD analysis, Fig. 1a shows the XRD patterns of all three samples under study. Both highly defective SnS_2_/HGF and moderately defective SnS_2_/HGF agree well with the Berndtite-2 T crystalline phase of SnS_2_ (JCPDS No. 23-0677), indicating a pure SnS_2_ phase regardless of the amount of thiourea presented in the synthesis system (Fig. 1a, left). Noticeably, the diffraction peaks of highly defective SnS_2_/HGF are significantly broadened, indicating structural disordering and/or the presence of small crystallites in highly defective samples. According to the relative intensity of the peaks due to the diffraction of the (100) and (101) planes, it can be concluded that highly defective SnS_2_/HGF is dominated by the (101) exposed facets. In contrast, moderately defective SnS_2_/HGF is dominated by the (100) exposed facets. This also shows that an excessive amount of the sulphur reagent can hinder the crystal growth of SnS_2_ along the (001) facets. This can result in different electrochemical properties, confirmed in the later sections of the article. The XRD pattern of the SnS/HGF sample in Fig. 1a, right shows an orthorhombic SnS phase (JCPDS No. 39-0354), confirming that the thermal treatment of SnS_2_ gives SnS.

Raman spectroscopy is carried out on the same samples (Fig. 1b). In Fig. 1b, left, the Raman spectra of the highly defective SnS_2_/HGF and moderately defective SnS_2_/HGF depict peaks at 314 and 205 cm^−1^, attributed to the characteristic out-of-plane vibration *A*_1g_ and in-plane vibration *E*_g_ modes in SnS_2_ [35, 36]. The Raman modes originate from the vibrations of chemical bonds, and therefore, different crystal facets contribute to different Raman-active modes. The *A*_1g_/*E*_g_ ratio difference indicates different exposed facets, which agrees well with the XRD results. The Raman spectrum of the SnS/HGF (Fig. 1b right) also confirmed the SnS phase because of the Raman peaks at 188, 220, and 161 cm^−1^, assigned to the *A*_g_ and *B*_3g_ modes in SnS [37].

Figure 1c (the left panel) shows the Sn 3*d* XPS spectra of the highly defective SnS_2_/HGF and defect-free SnS/HGF samples. It is seen that the positions of the two Sn 3*d* peaks of SnS/HGF shifted towards the lower binding energy with 0.8 eV difference in comparison to that of highly defective SnS_2_/HGF, indicating a lower oxidation state of Sn in SnS/HGF. The S 2*p* spectrum of sample highly defective SnS_2_/HGF (Fig. 1c, the right panel) shows two major peaks at 161.8 eV (S 2*p*_3/2_) and 163.0 eV (S 2*p*_1/2_), respectively. For SnS/HGF, the asymmetrical S 2*p* peaks were divided into three doublets, indicating three different chemical environments of S, namely Sn (IV)-S (161.8 eV for S 2*p*_3/2_), Sn (II)-S^2−^ (161.1 eV for S 2*p*_3/2_), and S–S (163.7 eV for S 2*p*_3/2_). This is predicted to be caused by the SnS_2_ dissociation and sulphur depletion during the thermal treatment process, which produced SnS with S residue. Overall, the XPS results agree well with the earlier XRD and Raman results, confirming the rational design of SnS_*x/*_HGF composites with highly defective, moderately defective, and defect-free 2D SnS_*x*._

**Fig. 2:**
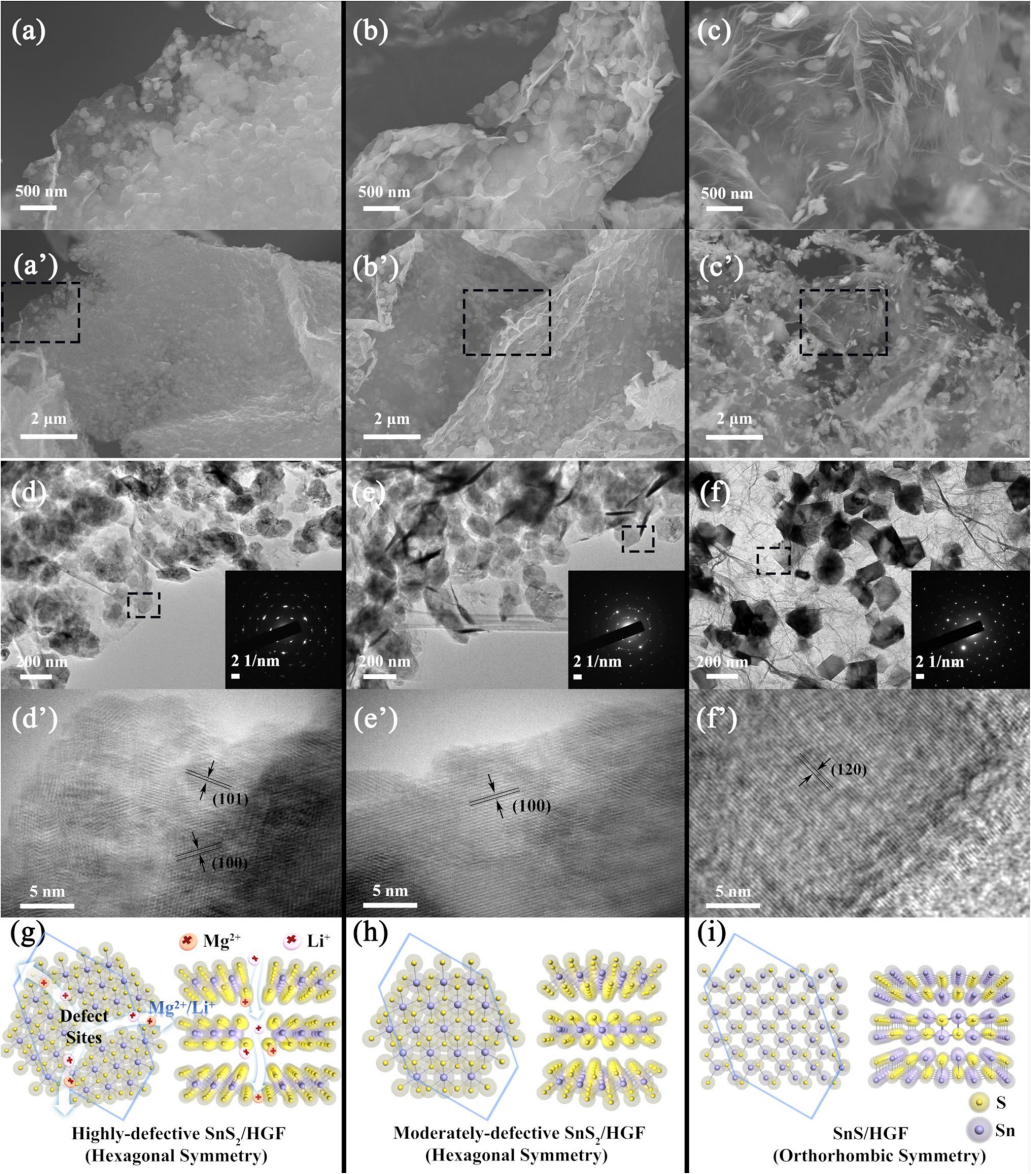
Morphology analysis of SnS_*x*_/HGF. FESEM images of (**a, a’**) highly defective SnS_2_/HGF, (**b, b’**) moderately defective SnS_2_/HGF, and (**c, c’**) defect-free SnS/HGF at different magnifications. The SEM images of HGF and neat SnS_*x*_ with various magnifications are shown in Figure S1-2. HRTEM images of (**d, d’**) highly defective SnS_2_/HGF, (**e, e’**) moderately defective SnS_2_/HGF, and (**f, f’**) SnS/HGF at different magnifications. Their respective selected area electron diffraction (SEAD) patterns of the samples are shown in the insets. (**g, h, i**) Crystal structures of the different corresponding SnS_*x*_/HGF viewed along with the crystallographic c- and a-axis

Figure S1 shows the morphology of the HGF. Such 3D structure features are favourable for charge transport in the electrochemical processes. Additionally, this 3D architecture can stabilize the SnS_*x*_ nanosheets and suppress self-aggregation against electrochemical cycling commonly occurring in SnS_*x*_ nanosheets as shown in Fig. S2. Figure 2a–c and a’–c’ shows that the SnS_*x*_ nanosheets are confined in the HGF. These results indicate that incorporating HGF to SnS_*x*_ also mediates the growth of the SnS_*x*_. It is important to note that the presence of HGF during SnS_*x*_ formation does not interfere the nucleation and crystal growth of 2D SnS_*x*_ sheets, as confirmed by the XRD results in Fig. S3.

Figure 2d–f and d’–f’ shows the HRTEM of SnS_*x*_/HGF. The lateral sizes of the SnS_*x*_ sheets in the samples are between 50 and 200 nm. The selected area electron diffraction (SAED) patterns (the insets in Fig. 2d–f) confirm SnS_2_ with hexagonal symmetry and SnS with orthorhombic symmetry. The HRTEM image of SnS_2_/HGF also shows numerous nanocrystals with abundant defective sites at grain boundaries (Fig. 2d’). The appearance of two different lattice fringes with interlayer distances of 3.16 and 2.78 Å in the highly defective SnS_2_/HGF is assigned to the (100) and (101) planes of Berndtite-2 T type SnS_2_, respectively. In comparison, continuous lattice fringes with an interlayer distance of 3.16 Å can be observed from the moderately defective SnS_2_/HGF (Fig. 2e’). The absence of nanodomains confirms that the excessive thiourea in the hydrothermal synthesis system was a prerequisite for forming defective SnS_*x*_. The HRTEM image of sample SnS/HGF in Fig. 2f’ shows fine fringes of SnS with a long-distance ordering. The interatomic distances were determined to be 3.4 Å, corresponding to the (120) plane of orthorhombic SnS.

As illustrated in Scheme 1, nanodomains in highly defective SnS_2_/HGF provide sufficient grain boundaries and interfacial regions. This structure can significantly increase the exposure of active edge sites towards electrolyte ions and subsequently form vacancy-like sites, which can provide percolation pathways, facilitating the diffusion of the ions [38, 39]. This is of significance given Mg^2+^ storage suffers a strong electrostatic repulsion [11]. Moreover, the moderate disorder with remaining periodicity and interdomain S–Sn–S electron conjugation on basal planes can improve electron conductivity.

### Electrochemical Performance

**Fig. 3:**
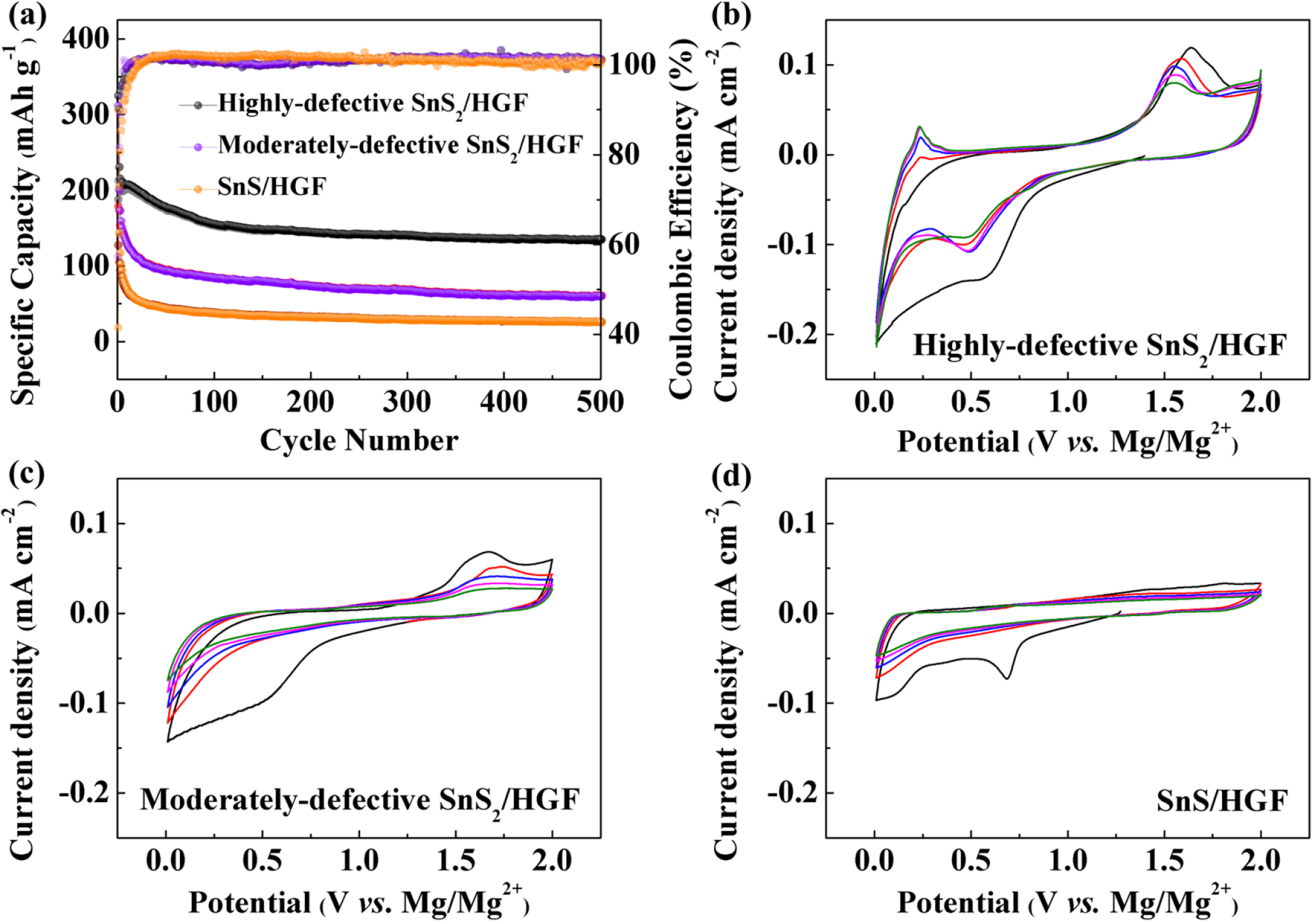
The electrochemical performance of the SnS_*x*_/HGF cathode materials in MIBs in the voltage window between 0.01 and 2.0 V versus Mg^2+^/Mg. **a** cycling performance at 50 mA g^−1^, **b** CV curves at 0.2 mV s^−1^ of highly defective SnS_2_/HGF, **c** moderately defective SnS_2_/HGF, and **d** SnS/HGF

The SnS_*x*_/HGF are now electrochemically evaluated as MIB and MLHB cathodes. The MIBs and MLHBs are first assembled in a 2032-type coin cell with the SnS_*x*_/HGF composite as the cathode, magnesium ribbon as the anode, and Celgard 3501 as the separator. The APC electrolyte is utilized in the MIBs, and a dual-salt electrolyte containing both APC and lithium chloride is used in the MLHBs. First, with MIBs, the deep cycling performance of SnS_*x*_/HGF is carried out at a current density of 50 mA g^−1^, and the cyclic voltammograms (CV) curves are recorded at a scan rate of 0.2 mV s^−1^. As shown in Fig. 3a, the specific capacity from highly defective SnS_2_/HGF electrode reached over 200 mAh g^−1^ and remained around 145 mAh g^−1^ after 200 cycles. This cycle performance outperforms moderately defective SnS_2_/HGF (75 mAh g^−1^ after 200 cycles) and SnS/HGF (30 mAh g^−1^ after 200 cycles) electrodes. The higher electrochemical performance from highly defective SnS_2_/HGF indicates that the defect-rich structure is beneficial for the reaction activity towards Mg^2+^. Also, we note that further quantitative analysis of charge storage mechanisms in the electrochemical process of the three SnS_*x*_/HGF electrodes revealed that moderately defective SnS_2_/HGF and SnS/HGF had the most capacity contributed from capacitive reactions at the surface as compared to highly defective SnS_2_/HGF. Related deep analysis is given in the last section (*i.e..,* charge transport kinetics) of the article. Figure 3b–d shows the CVs of the three electrodes in the potential window of 0.01–2.0 V. The large area of the CV curve obtained from highly defective SnS_2_/HGF represents its higher capacity in comparison to moderately defective SnS_2_/HGF and SnS/HGF. To account for the observed electrochemical differences, the CV curves of each sample are analysed. First, for highly defective SnS_2_/HGF, the pair of peaks at 0.5 V in the cathodic scan and 1.5 V in the anodic scan correspond to the conversion reaction proposed as SnS_2_ + *x*Mg^2+^ + 2*x*e^−^ ↔ MgS + Sn. During substantial cycling (Fig. 3b), a new anodic peak at around 0.25 V appeared, attributed to de-alloying reaction from Mg_2_Sn to Sn, according to the previous report [40]. As a result, the corresponding alloying reaction should occur at around 0.1 V (Sn + 2Mg^2+^ + 4e^−^ ↔ Mg_2_Sn). For the moderately defective SnS_2_/HGF sample, the CV peaks corresponding to the conversion reaction around 0.5 and 1.5 V decrease significantly in the first few cycles, indicating reduced redox capacities and inferior electrochemical performance. On the other hand, the CV curves of SnS/HGF show a capacitance behaviour, in which no significant peak can be observed except the first cathodic scan. Compared with the other two samples, the significantly enlarged peak current of highly defective SnS_2_/HGF indicates that defect-rich structure is beneficial for the reaction activity towards Mg^2+^. In comparison, the negligible peaks in CV curves of moderately defective SnS_2_/HGF and SnS/HGF indicate that their capacity contribution is mostly from capacitive reactions at the surface. This result was consistent with the electrochemical capacity differences shown in Fig. 3a. Galvanostatic charge and discharge curves of SnS_*x*_/HGF cathodes in MIBs are shown in Fig. S4. At current density 50 mA g^−1^, all samples show relatively smooth voltage profiles due to the sluggish Mg^2+^ insertion kinetics. However, compared to the sloping curves of the moderately defective SnS_2_/HGF and SnS/HGF samples, a small discharge plateau at around 0.5 V and charge plateaus at around 0.25 and 1.5 V, respectively, can be seen from the highly defective SnS_2_/HGF, in accordance with the CV results in Fig. 3b.

**Fig. 4:**
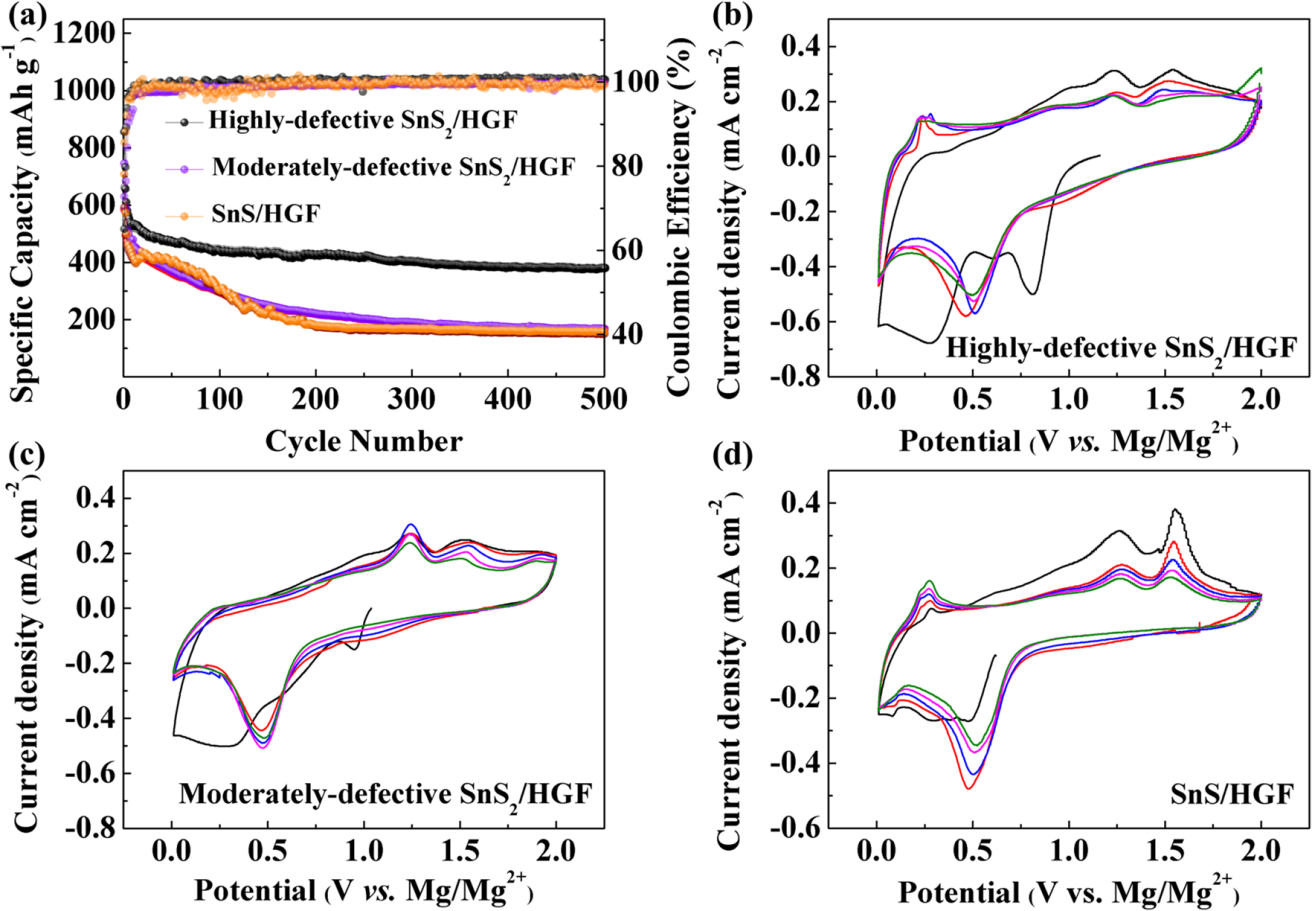
Electrochemical properties of highly defective SnS_2_/HGF, moderately defective SnS_2_/HGF and SnS/HGF in MLHBs between 0.01–2.0 V vs. Mg^2+^/Mg. **a** Stability at 50 mA g^−1^. **b, c, d** CV curves at 0.2 mV s.^−1^

Figure 4 shows the cycling stability and CV curves of SnS_*x*_/HGF cathodes in MLHBs. Similar to MIB results, highly defective SnS_2_/HGF delivers a higher specific capacity than the moderately defective SnS_2_/HGF and SnS/HGF, with SnS/HGF giving the least specific capacity. To be specific, highly defective SnS_2_/HGF specific capacity can be seen to reach 600 mAh g^−1^ at the 3rd cycle, which remains relatively stable to a tune of 430 mAh g^−1^ after 200 cycles at a low current density of 50 mA g^−1^. The moderately defective SnS_2_/HGF delivers a slightly reduced capacity of around 540 mAh g^−1^ at the 3rd cycle and further decays to 220 mAh g^−1^ after 200 cycles at the same current density. On the other hand, SnS/HGF delivers a stable cycle performance in the first 50 cycles with a specific capacity of around 420 mAh g^−1^. However, a rapid capacity decay to around 180 mAh g^−1^ after 200 cycles is observed. This capacity change of the SnS/HGF electrode results from the structure distortion or activation process during the insertion of foreign ions [40]. The superior capacity and stability of highly defective SnS_2_/HGF can be attributed to two factors: (1) the enlarged accessible surface with active sites and (2) the defective structure in which the vacancies can buffer structural changes during ion insertion [41, 42].

Figure 4b–d shows the CV curves of the SnS_*x*_/HGF cathodes obtained from MLHBs. In general, SnS_*x*_/HGF cathodes in MLHBs show obvious cathodic/anodic peaks and larger enclosed areas than that in MIBs, suggesting enhanced charge storage performance in the former. This can be attributed to the additive LiCl, which enhances the conductivity of the hybrid electrolyte, and the Cl^−^ anion diminishes the interface resistance of Mg deposition/dissolution by destroying the blocked solid electrolyte interphase (SEI) on the Mg anode [14, 21]. For the highly defective SnS_2_/HGF and moderately defective SnS_2_/HGF CV (Fig. 4), the first cathodic scan shows several peaks below 1 V. The peak around 0.9 V corresponds to the phase transformation from SnS_2_ to Li_*x*_SnS_2_ due to Li^+^ insertion (SnS_2_ + *x*Li^+^ + *x*e^−^ → Li_*x*_SnS_2_) [43]. The irreversible broad peak in the low-voltage area was due to the SEI [14]. In the subsequent cycles, the reversible cathodic peak around 0.5 V is attributed to conversion reactions between SnS_2_ and Mg^2+^ (SnS_2_ + *x*Mg^2+^  + 2*x*e^−^ → MgS + Sn), and SnS_2_ and Li^+^ (Li_*x*_SnS_2_ + (4-*x*)Li^+^  + (4-*x*)e^−^ → Sn + 2Li_2_S, where 0 < *x* ≤ 2) [26]. The additional anodic peak at 1.2 V is assigned to the reversible conversion reaction involving Li_2_S decomposition [44]. In comparison, the anodic peak at around 1.5 V corresponds to the reversible conversion reaction involving MgS decomposition, similar to that in highly defective SnS_2_/HGF in MIBs (Fig. 3). In addition, the reversible anodic peak around 0.1 V and the emerging reversible cathodic peak around 0.25 V is due to the Sn–Mg alloying/de-alloying reactions (Sn + 2Mg^2+^ + 4e^−^ ↔ Mg_2_Sn) [45]. All of the reaction peaks can also be observed from the galvanostatic discharge and charge curves of SnS_*x*_/HGF cathodes in MLHBs as shown in Fig. S5. It is important to note that the redox potential of Mg^2+^/Mg is higher than that of Li^+^/Li, and the typical alloying reaction between Sn and Li^+^ is in the range of 0.1–0.5 V versus Li^+^/Li [46]. Therefore, the Sn-Li alloying reaction can be ruled out in hybrid batteries. Different from highly defective SnS_2_/HGF, the de-alloying peak around 0.25 V is not obvious in the CV curve of moderately defective SnS_2_/HGF. This limited Sn-Mg alloying reaction should be caused by the sluggish Mg diffusion kinetics in moderately defective SnS_2_/HGF. As shown in Fig. 3, the moderately defective SnS_2_/HGF shows negligible capacities towards Mg^2+^. Thus the reaction between Li^+^ and moderately defective SnS_2_/HGF is expected to dominate the hybrid batteries. At the anode side, Mg^2+^ is reversibly and preferentially plated back to the Mg anode, and the co-deposition of Mg^2+^ and Li^+^ ions to form Mg-rich Mg-Li alloy cannot be ruled out [47]. Figure 4d shows the CV curve of SnS/HGF cycled in hybrid batteries, where similar peaks representing conversion reactions (SnS + 0.5*x*Mg^2+^ + *x*e^−^ → MgS + Sn, Li_*x*_SnS_2_ + (4-*x*)Li^+^ + (4-*x*)e^−^ → Sn + 2Li_2_S (0 < *x* ≤ 2)), and alloying reaction (Sn + 2Mg^2+^ + 4e^−^ ↔ Mg_2_Sn) are also observed. Moreover, peak intensity decays at around 1.5 V in the other curves for all the samples. This phenomenon is predicted to stem from the dissolution of S-based species [19], indicating the partially irreversible SnS_*x*_ (*x* = 1 or 2) reformation.

Published literature shows that it is possible to realize a fully or partially reversible conversion of SnS_2_ in LIBs [48, 49]. Nevertheless, these reactions are difficult to confirm because of the irreversible electrochemical amorphization. Collectively, it can be concluded that the reaction of SnS_*x*_/HGF in Mg^2+^/Li^+^ hybrid battery involves the co-insertion of both Mg^2+^ and Li^+^, which undergoes the conversion reaction and the subsequent alloying reaction. Unlike the systems characterized by single-electron transfer at the cathode, the multi-electron transfers based on conversion and alloying reaction are simultaneously satisfied at both the electrodes in this work. The observed excellent electrochemical performance surpasses the previously reported hybrid battery systems based on intercalation-type cathode materials under comparable rates and Li salt concentration (Table S1) [13–16].

**Fig. 5:**
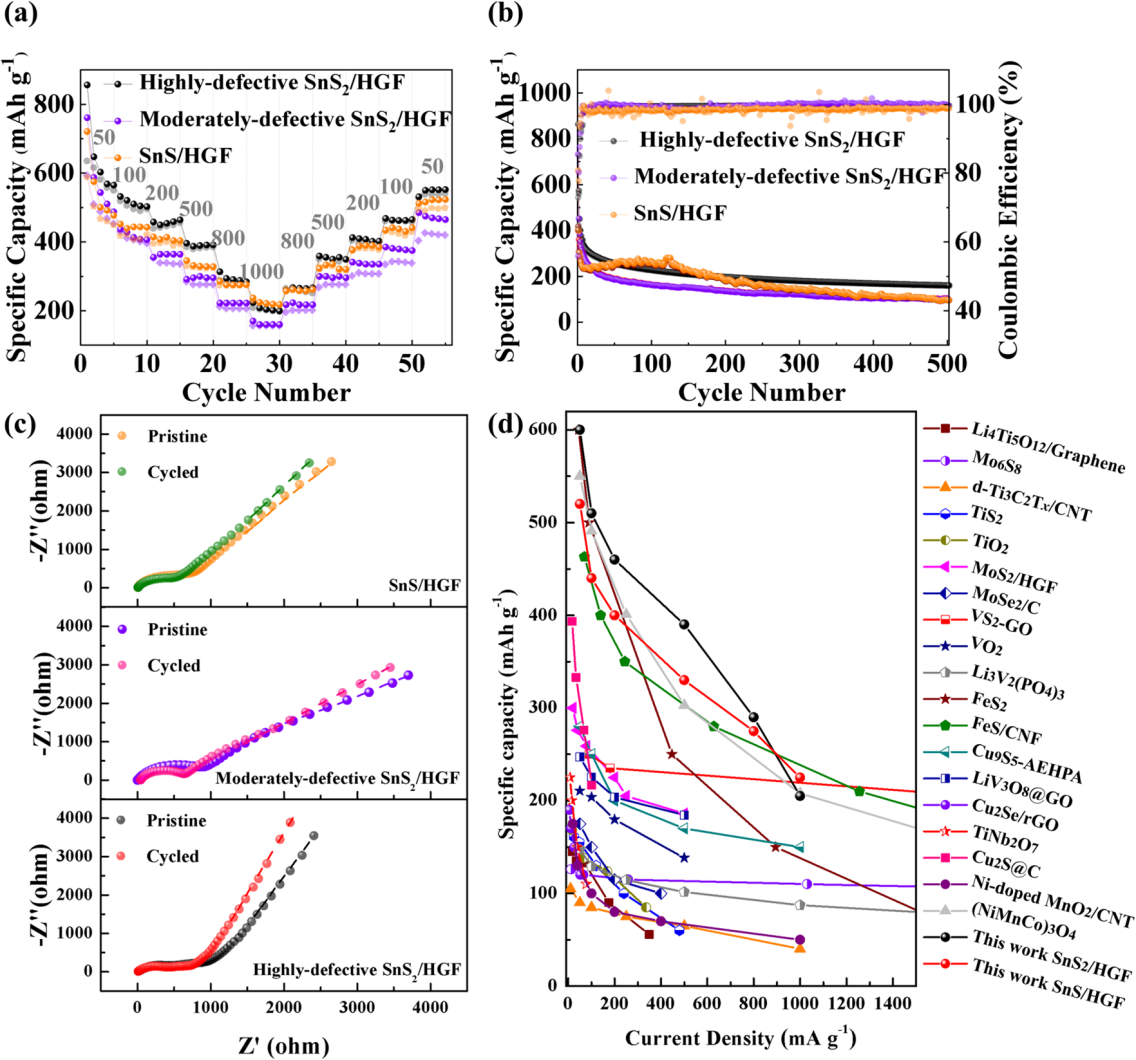
Electrochemical performance of highly defective SnS_2_/HGF, moderately defective SnS_2_/HGF, and defect-free SnS/HGF in MLHB. **a** rate performance, **b** cycling stability at 800 mA g.^−1^, **c** Nyquist plots at the OCP before and after cycling (the dotted lines are fitting curves using the equivalent circuit shown in Fig. S7h), and **d** rate capability in comparison with those reported in the literature [13–16]

Capacity retention over a range of scan rates and long-cycle performance of SnS_*x*_/HGF cathodes in MLHBs are investigated next. Experimental results show that highly defective SnS_2_/HGF and SnS/HGF have excellent reversibility after high-rate cycling, whereas the that of moderately defective SnS_2_/HGF is inferior (Fig. 5). The reversible specific capacities of the highly defective SnS_2_/HGF reached 600, 510, 460, 390, 290, and 205 mAh g^−1^ at the current density of 50, 100, 200, 500, 800, and 1000 mA g^−1^, respectively, indicating a good rate capability. In the case of defect-free SnS/HGF, the specific capacity only reached 520, 440, 400, 330, 275, and 225 mAh g^−1^ at the same current densities and recovered to ~ 500 mAh g^−1^ (~ 96% retention) after the current density returns to 50 mA g^−1^. For the moderately defective SnS_2_/HGF electrode, it delivered specific capacities of 540, 415, 364, 295, 222, and 159 mAh g^−1^ at the above current densities, respectively, and about 87% the capacity retention was obtained when the current density was changed back to 50 mA g^−1^. The highly defective SnS_2_/HGF displayed the best long-term cycling stability at 800 mA g^−1^ among the three samples with a reversible specific capacity of 160 mAh g^−1^ after 500 cycles (Fig. 5b). This good reversibility stems from the defect-rich structure, which can offer percolation pathways for efficient ion transport. In addition, the vacancy-like defective sites buffered structural changes caused by Mg^2+^ and Li^+^ insertion.

It is interesting to note that the specific capacity of SnS/HGF-based MLHB firstly increased, then decreased (Fig. 5b). We believe this is related to the perfect structure of SnS without defective sites. The insertion of Li^+^ and Mg^2+^ (particularly the latter) needs time to activate the layered structure. As a result, the capacity change of the SnS/HGF electrode gradually increased in the first few cycles. The insertion also gradually caused structure damages and/or collapses; thus, the capacity gradually decreased after the material was fully activated.

The experimental results shown in Figs. 4a and 5b demonstrate that the highly defective SnS_2_/HGF sample displayed an excellent stability against cycling during 500 cycles, and outperformed the moderately defective SnS_2_/HGF sample. These observations indicate that the introduction of defects does not affect the strength of the entire SnS_*x*_/HGF cathode. This can be explained by the following reasons: (1) The nanosize SnS_x_ (Fig. 2d) can alleviate volume expansion; (2) the highly defective SnS_2_/HGF has grain boundaries, which can provide percolation pathways, and play a buffering role in volume change during cycling; and (3) the holey graphene foams stabilize the SnS_*x*_ nanosheets and suppress self-aggregation against electrochemical cycling.

Additionally, SnS/HGF delivers better performance at high rates than that of moderately defective SnS_2_/HGF even without the defect-rich environment. More notably, its specific capacity retention of 96% after the rate capability test is higher than that of all the defective SnS_x_/HGF cathodes. We explain these observations as follows; (1) compared to SnS_2_, the conversion reaction of SnS has a more straightforward phase transformation process without the participation of the disruption/recombination of the extra (S–S)^2−^ bond [50, 51]; (2) since one Mg^2+^ carries two charges, electron transport efficiency becomes more important in Mg-based batteries, where the electrode with poor electron conductivity will remarkably lead to poor performance at high rates. We confirm this with electrochemical impedance spectroscopy (EIS), in which we observe SnS/HGF having a lower charge-transfer resistance than defective SnS_*x*_/HGF (Fig. 5c) [52]. This is true as SnS has higher crystallinity than SnS_2_ and thus favours fast electron transport, which translates into better high-rate performance in the cathodes; (3) beyond the different ohmic contributions of the studied SnS_*x*_/HGF, the different phases of tin (*α*-tin and *β*-tin) produced by SnS and SnS_2_ during cycling also can affect the electrode performance [53]. As investigated by Im et al. [45], when the orthorhombic-phase SnS was cycled in the presence of Li^+^, *α*-tin with a diamond cubic crystal structure became the main phase with the increasing cycle numbers. In contrast, in the case of SnS_2_, the main Sn phase after cycling became metallic *β*-tin with a tetragonal crystal structure. These two instances tend to offer different metal ion diffusion barriers. For example, Wang et al. [53] proved that the diffusion barrier of Mg^2+^ in *α*-tin is smaller than that in *β*-tin. Therefore a better high-rate performance can be obtained from SnS than with SnS_2_.

To decouple the enhanced performance contributed by the HGF conductive open structure, we also investigate the electrochemical performance of HGF and pure SnS_*x*_ without HGF. As shown in Figs. S6 and S7, the capacity contribution from HGF is negligible, and it is evident that the charge-transfer resistance of SnS_*x*_/HGF was reduced due to the presence of HGF. Also, SnS_*x*_/HGF electrodes also show enhanced rate performance compared to pure SnS_*x*_ samples. Figure 5d shows the rate capability of the SnS_*x*_/HGF electrodes in comparison to those reported in the literature [13–16]. The graph reveals that our SnS_*x*_/HGF electrodes have excellent electrochemical performance compared to the previously reported hybrid batteries based on insertion cathodes at comparable rates.

The cycle performance of highly defective SnS_2_/HGF in LIB with LiPF_6_-EC-DEC as the electrolyte is compared with that in MLIB with APC and LiCl as the electrolyte in Fig. S8. Considering that the redox potential of Mg^2+^/Mg is 0.67 V higher than that of Li^+^/Li, the voltage window of LIB was limited between 0.8 and 2.8 V that is equivalent to the voltage range of 0.01–2.0 V versus Mg^2+^/Mg used in MLIBs [13–16]. It can be seen from Fig. S8 that the capacities at different rates in LIBs are higher than that in MLIBs in the first few cycles. However, the cycling stability of the highly defective SnS_2_/HGF in MLIBs is better than that in LIBs at both low and high rates. This may be due to the structure distortion caused by a more rapid Li^+^ insertion/de-insertion kinetics than that of the Mg^2+^ case.

### Phase Evolution During Cycling: Verification of Mg^2+^ and Li^+^ Insertion in SnS_***x***_/HGF

The ex situ XPS study of Mg 2*p* and Li 1*s* for fully discharged SnS_*x*_/HGF cathodes at 0.01 V versus Mg^2+^/Mg is employed to verify the insertion of Mg^2+^ in MIBs and co-insertion of Mg^2+^ and Li^+^ in MLHBs. In MIBs, we observe a peak shift to the higher binding energies of highly defective SnS_2_/HGF compared to the less defective samples, indicating a different insertion state of Mg^2+^ (Fig. S9a). This observation means that the nature of the existence of magnesium in highly defective SnS_2_/HGF is different from that of its counterparts. In moderately defective SnS_2_/HGF and SnS/HGF with Mg 2*p* peak at ~ 50.4 eV, the magnesium in these cathodes is absorbed Mg^2+^ without chemical bonding, whereas in highly defective SnS_2_/HGF with Mg 2*p* peak at ~ 51.0 eV, is both absorbed and bonded magnesium with S species of the cathode. In MLHBs, the co-insertion of Mg^2+^ and Li^+^ is also confirmed using the same technique in which both strong peaks of Mg 2*p* and Li 1*s* are observed (Fig. S9b). We also note that the percentage of inserted Mg compared to Li is also different in highly defective SnS_2_/HGF (Figs. S9b and S10 inset table). The Mg inserted for highly defective, moderately defective SnS_*x*_/HGF and defect-free SnS/HGF was 37.7%, 24.1%, and 29.4%, respectively. Whereas the Li inserted for highly defective, moderately defective SnS_x_/HGF and defect-free SnS/HGF was 62.3%, 75.9%, and 70.6%, respectively. The highly defective cathode gives higher Mg^2+^ insertion than those with lower defects, suggesting that the defect-rich structure is critical in promoting Mg^2+^ insertion. This confirms that the highly defective SnS_2_/HGF cathode can realize the co-insertion of Mg^2+^ and Li^+^ while bypassing the requirement for a large amount of electrolyte solvent with Li^+^ salt. In addition, as the prevailing Li^+^ insertions can reduce the insertion barrier of the following Mg^2+^ insertion [13, 17], this Mg^2+^-Li^+^ co-insertion cathode can further improve the utilization of Mg^2+^ ions and the electrochemical performance of MLHBs.

**Fig. 6:**
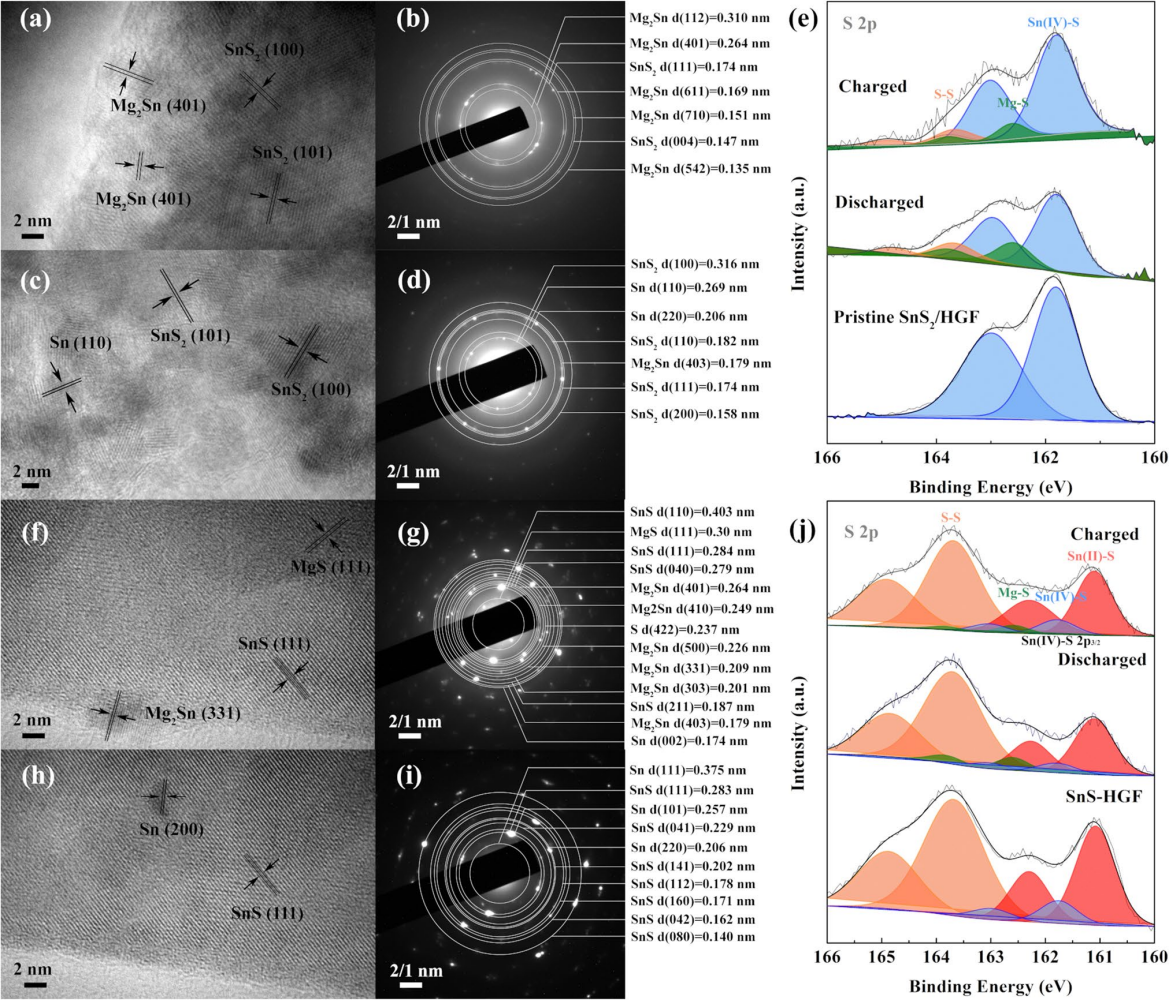
Ex situ HRTEM images and corresponding SAED pattern in MIBs. **a, b** highly defective SnS_2_/HGF and **f, g** SnS/HGF after fully discharged to 0.01 V. **c, d** highly defective SnS_2_/HGF and **h, i** SnS/HGF after being charged back to 2.0 V. Ex situ XPS spectra represent valence changes of S 2*p* of the **e** highly defective SnS_2_/HGF and **j** SnS/HGF after the first cycle at charged and discharged stages

Furthermore, the local microstructural changes and phase evolution of the highly defective SnS_2_/HGF after the first cycle in both MIBs and MLHBs are investigated using ex situ high-resolution XPS and HRTEM. In addition, SnS/HGF is also evaluated due to the different S coordination numbers. First, Fig. 6 shows the corresponding structure results from cycled highly defective SnS_2_/HGF and SnS/HGF electrodes with MIBs. At fully discharged stage, the HRTEM image and the corresponding SAED patterns of highly defective SnS_2_/HGF (Fig. 6a–b) shows fringes assigned to Mg_2_Sn, demonstrating the involvement of conversion reactions (SnS_2_ + *x*Mg^2+^ + 2*x*e^−^ → Sn + 2MgS) and subsequent alloying reactions (Sn + 2Mg^2+^ + 4e^−^ ↔ Mg_2_Sn). On the other hand, the discharged SnS/HGF shows well-preserved lattice fringes assigned to SnS along the planar direction with some small crystals related to Mg_2_Sn (Fig. 6f-g).

Upon charging, the corresponding HRTEM image and SAED patterns of highly defective SnS_2_/HGF (Fig. 6c and d) show that the lattice fringes of SnS_2_ and defects on the planar direction are preserved after cycling. Also, the appearance of the metallic Sn phase in the charged stage suggests reversible alloying reactions in highly defective SnS_2_/HGF. Meanwhile, the original long-distance orderings of SnS/HGF at the charged stage are also well maintained without any obvious changes (Fig. 6h), implying that no major phase change took place. The SAED pattern of charged SnS/HGF indicates that the major phase was SnS (Fig. 6i). Collaborative analysis from XPS of highly defective SnS_2_/HGF and SnS/HGF at different charge/discharge stages confirms the results above. At the fully discharged stage, new S 2*p* peaks assigned to S–S (163.7 eV for S 2*p*_3/2_) and MgS (162.6 eV for S 2*p*_3/2_) of highly defective SnS_2_/HGF are observed (Fig. 6e). In contrast, no obvious changes can be observed in the SnS/HGF electrodes, indicating no noticeable phase change during the electrode cycling (Fig. 6j). As a result, the ion storage mechanism of SnS/HGF in MIBs should be dominated by the capacitive adsorption of charges, a conclusion reconfirmed by kinetics analyses in Fig. S11.

**Fig. 7:**
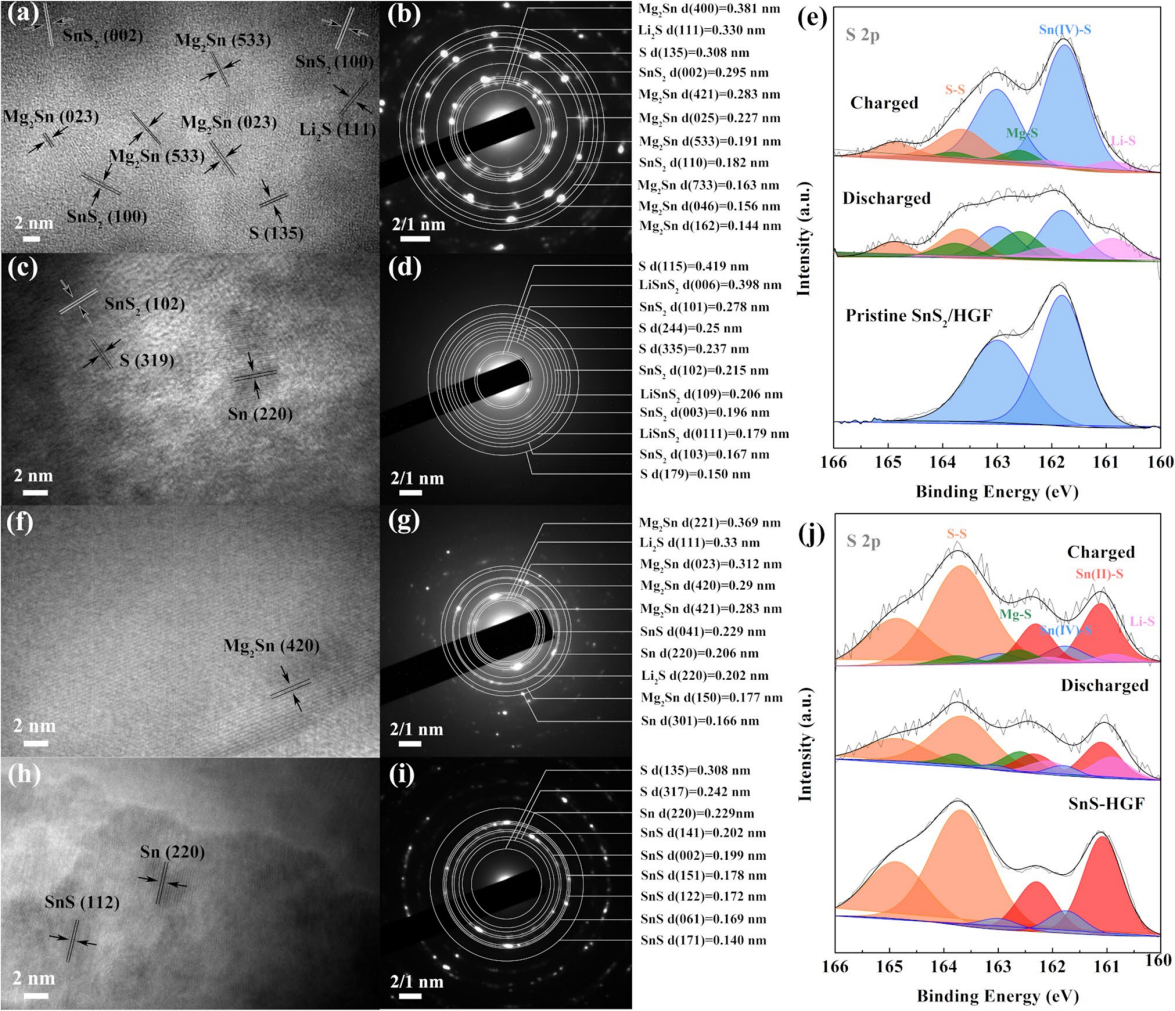
Ex situ HRTEM images and corresponding SAED pattern in MLHBs. **a, b** highly defective SnS_2_/HGF and **f, g** SnS/HGF after fully discharged to 0.01 V. **c, d** highly defective SnS_2_/HGF and **h, i** SnS/HGF after charged back to 2.0 V. *Ex-situ* XPS spectra represent valence changes of S 2*p* of the **e** highly defective SnS_2_/HGF and **j** SnS/HGF after the first cycle at charged and discharged stages

Next, the local microstructural changes and phase evolution of the highly defective SnS_2_/HGF and SnS/HGF after the first cycle in MLHBs are probed. As depicted in Fig. 7a, after the first discharge, the pristine SnS_2_ in highly defective SnS_2_/HGF evolved into nanodomains assigned to Mg_2_Sn. The presence of Li_2_S and S is also confirmed in SAED results (Fig. 7b), indicating a Li-driven conversion reaction and subsequent Mg-Sn alloying reaction from SnS_2_ to Mg_2_Sn and Li_2_S. The existence of MgS representing the conversion reaction between Mg^2+^ and SnS_2_ is confirmed by the XPS results, in which new S 2*p* peaks assigned to MgS (162.6 eV for S 2*p*_3/2_) are observed (Fig. 7e). When charged back, numerous nanoclusters (nanoparticle-in-matrix) are generated, forming a porous network (Fig. 7c). According to the SAED patterns, these nanodomains mainly consisted of S, SnS_2,_ and LiSnS_2_ phases (Fig. 7d). As for SnS/HGF, the HRTEM and corresponding SAED results in Fig. 7f–g confirm the main phase Mg_2_Sn and other phases of Sn and Li_2_S at the fully discharged state. The existence of MgS (162.6 eV for S 2*p*_3/2_) and S (163.7 eV for S 2*p*_3/2_) is also confirmed in SAED (Fig. 7j). When charged back, the SnS/HGF displays the SnS and Sn phases from the de-magnesiated Mg_2_Sn (Fig. 7h–i).

Different from the XPS results from the MIBs, in the MLHBs, the main S 2*p* peaks of both highly defective SnS_2_/HGF and SnS/HGF cathodes show notable changes (Fig. 7e–j). After being discharged to 0.01 V, the peaks associated with Sn-S from both the samples are considerably weak. In addition, new peaks assigned to MgS (162.6 eV for S 2*p*_3/2_) and Li–S (160.9 eV for S 2*p*_3/2_) [54, 55] appeared, indicating a conversion reaction. Moreover, the percentage of MgS peaks is higher in MLHBs than in MIBs, indicating that the lithiation process promotes the subsequent conversion reactions involving Mg^2+^. The intermediate phase evolves into the final discharge products of tin alloys and lithium/magnesium sulphides. When the battery is charged back, the remarkably attenuated Li–S bonds compared to the previous discharged samples confirm the reversibility of the conversion reaction of S. Furthermore, the peaks corresponding to MgS of the charged products in MLHBs are reduced compared with that in MIBs. This observation confirms better reversibility of the conversion reaction of sulphur in the hybrid battery than in pure MIBs. This is because the Lewis acid Li^+^ can play an active role in enhancing the reversibility of the Mg/S system, either by coordinating with the surface S^2−^ of MgS to increase its solubility [56], or by lithiating MgS into rechargeable hybrid Mg/Li polysulphide (Mg-Li-S) [57]. Tao et al. [58] have also confirmed the assisting effect of Li^+^ on Mg/S reversibility by Mg metal corrosion experiments. Therefore, the addition of Li^+^ promotes the phase transformation of SnS_*x*_ (*x* = 1 or 2) and enhances the reversibility of generated S. This is an important factor for bridging the gap between LiBs and MIBs in such hybrid batteries consisting of both metal ions.

### Charge Transport Kinetics

In conventional battery materials, the challenge lies in finding an effective way to enhance the reaction kinetics without sacrificing capacity. The highly defective SnS_2_/HGF sample showed high specific capacity with good rate performance. Therefore, apart from the influence of defects on the charge storage process during conversion and alloying reaction, the defects-induced capacitive contribution is further investigated by performing CV measurements at various scan rates from 0.2 to 4 mV s^−1^ in both MIBs and MLHBs (Figs. S11 and 8). The *b* values at different potentials *vs*. Mg^2+^/Mg are obtained by linear fitting plots of log *v *versus log *i*(*V*) according to *i*(*V*) = *av*^*b*^ [59], where *b* value of 0.5 represents a faradaic reaction controlled by semi-infinite linear diffusion, whereas a value of 1 indicates a surface-controlled process.

In MIBs (Fig. S11), during the cathodic scan, *b* values are close to 1 at a potential higher than 0.8 V, suggesting that the current response is predominantly surface controlled. When discharged to low potential (< 0.5 V), *b* values of all samples are close to 0.5, indicating the involvement of diffusion-controlled reaction process (conversion or alloying reactions) appearing around 0.5 V. The decreased *b* value of all samples indicates a gradual switching from capacitive process-dominated mechanism to dual contributions from diffusion and capacitive-controlled mechanisms. This transition reveals that a critical concentration of Mg^2+^ on the SnS_*x*_ surface has to be reached to trigger Mg^2+^ insertion. During the anodic scan, *b* values are decreased from near 1 at potential lower than 1 V to close to 0.5 at 1.5 V, corresponding to the Mg de-insertion from MgS around 1.5 V. However, considering the inferior cycling capacities (Fig. 3) and large capacitive contribution (93% in Fig. S11) of samples with few defects, it can be concluded that conversion or alloying reactions in these samples are difficult to be activated due to the limited active sites, or diffusion constraints of Mg^2+^. Thus, the dominated charge storage mechanisms for samples with few defects in MIBs are capacitive controlled, where the accumulated Mg^2+^ are prone to electrochemically combine at the surface. In contrast, the defect-rich environment of highly defective SnS_2_/HGF shows an improved charge storage capacity and favourable interactions with Mg^2+^ due to increased accessible active sites and sound Mg^2+^ diffusion brought about by the defects.

**Fig. 8:**
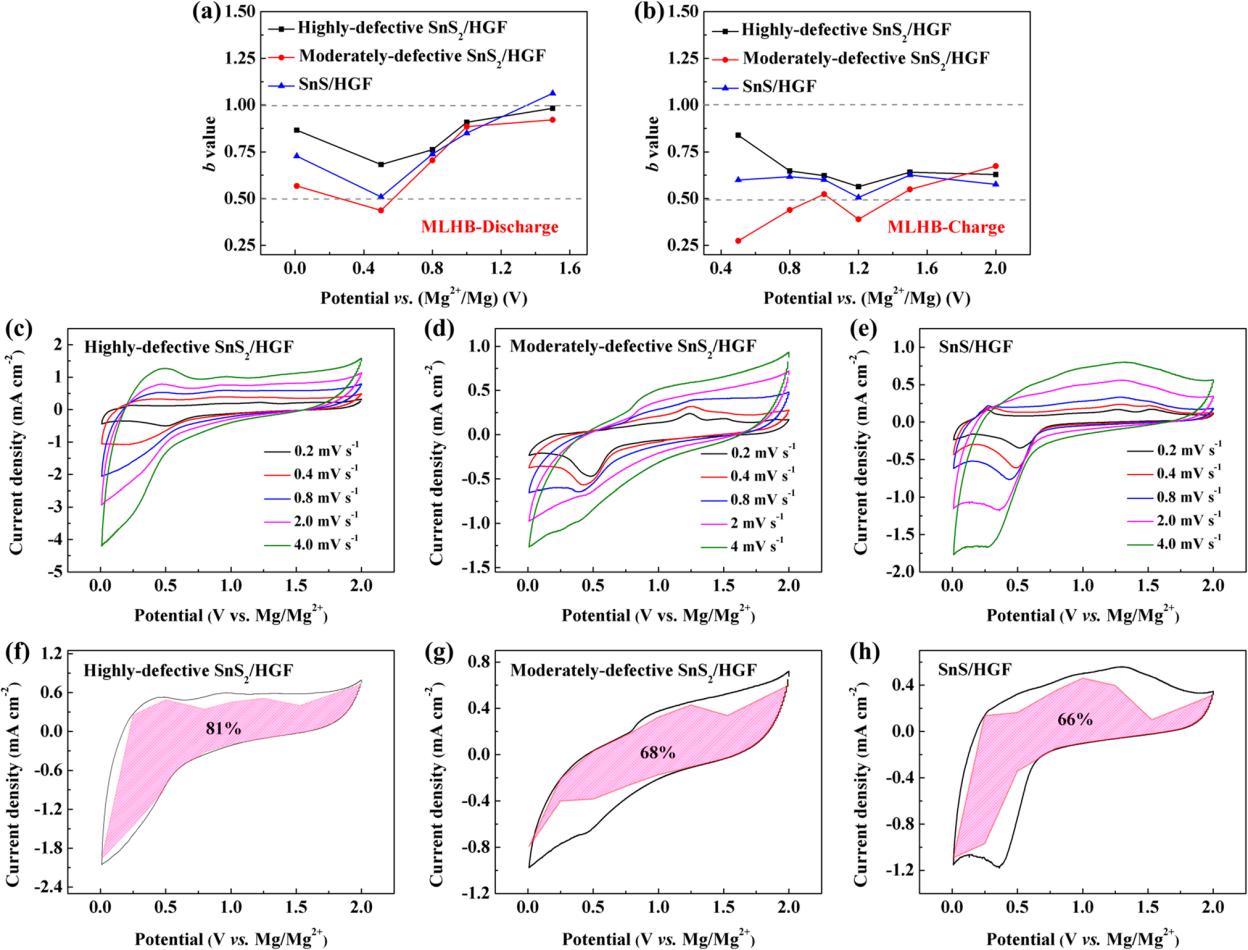
Kinetics and quantitative analysis of SnS_*x*_/HGF in MLHBs. *b* values of highly defective SnS_2_/HGF, moderately defective SnS_2_/HGF, and SnS/HGF at different potentials versus Mg^2+^/Mg. **a** During discharging and **b** charging processes. CV curves of **c** highly defective SnS_2_/HGF, **d** moderately defective SnS_2_/HGF and **e** SnS/HGF at various scan rates, and the corresponding capacitive charge storage contributions of **f** highly defective SnS_2_/HGF, **g** moderately defective SnS_2_/HGF and **h** SnS/HGF at a scan rate of 2.0 mV s.^−1^

In MLHBs, the calculated *b* values for cathodic and anodic peaks are between 0.5 and 1, signifying a mixture of diffusion-controlled and capacitive processes (Fig. 8a–b). *b* values of highly defective SnS_2_/HGF at most potentials are higher than the other two counterparts, signifying the good rate performance from significant capacitive contributions. When compared with the *b* values obtained from MIBs, different trends can be observed in MLHBs due to the participation of Li^+^. In cathodic scan, at 0.5 V (corresponding to the potential of conversion reaction), *b* values of samples with few defects are significantly decreased, whereas that of highly defective SnS_2_/HGF stayed at ~ 0.7. It is expected that the defect-induced capacitive-controlled charge storage can help to compensate for the relatively slow conversion reaction kinetics. At anodic 0.5 V, where the de-alloying reaction should be happening during the charging process, moderately defective SnS_2_/HGF gave the lowest *b* value. Apart from the limited active sites and diffusion channels compared with highly defective SnS_2_/HGF, the lowest *b* value of moderately defective SnS_2_/HGF comes from the different ohmic contributions of the samples themselves and different intermediate phases of conversion reaction (*α*-tin and *β*-tin) [53]. *b* values at anodic 1.2 V of all samples reached were the lowest due to the ongoing conversion reaction.

More still, the capacitive contribution to the current response is qualitatively measured by separating the current response (*i*) at a fixed potential (*V*) into the capacitive-like (*k*_1_*v*) and diffusion-controlled processes (*k*_2_v^1/2^) according to *i* (*V*) = *k*_1_*v* + *k*_*2*_*v*^1/2^ [59], where *k*_1_*v*, *k*_2_*v*^1/2^ represents the capacitive contribution and diffusion-controlled reaction contribution, respectively. By determining constants *k*_1_ and *k*_2_, we distinguish the fraction of the current at specific potentials from capacitive and diffusion-controlled effects. As shown in Fig. 8f–h, the typical voltage profiles of the capacitive currents are highlighted as the red region compared to the total current. For samples with few defects in MLHBs, the capacity percentage of conversion and alloying reaction are more compared to those in MIBs due to the co-insertion of Li^+^. However, the capacitive process is still a predominant charge storage mechanism in the highly defective SnS_2_/HGF, even in MLHB batteries. The capacitive contribution in highly defective SnS_2_/HGF reached a higher percentage (81% at 2.0 mV s^−1^) than those of the other two samples. The kinetic analysis revealed that the capacitive process is a predominant charge storage mechanism in the highly defective SnS_2_/HGF, resulting in fast ion storage kinetics without compromised capacities. The above analysis and discussions explain well why the defect-rich structure with many accessible active sites can offset the unfavourable Mg^2+^ diffusion enhancing charge storage with rapid ion storage kinetics, hence guaranteeing favourable interaction with Mg^2+^ to promote the Mg^2+^ involved conversion and alloying reactions. A trade-off between high-capacity and fast reaction kinetics is relatively resolved in such a scenario.

Another point worth noting is that the few defects in the moderately defective SnS_2_/HGF do help enhance the Mg^2+^ storage capacities in MIBs, but there was hardly any improvement in capacities of MLHB battery (Figs. 3 and 4). The main charge storage mechanism for the three samples in MIBs is electrocapacitive as revealed by the experimental results shown in Figs. 3b–d and S11. In addition, Fig. 3a shows that the cycling performance of the moderately defective SnS_2_/HGF sample is better than that of the defect-free SnS/HGF sample, indicating that the presence of defects in SnS_*x*_ improves the Mg^2+^ storage performance due to increased accessible active sites and improved Mg^2+^ diffusion kinetics. In MLHBs, the charge storage behaviour of the three samples is different. Figure 8 reveals that the electrocapacitive process is still dominant mechanism for charge storage mechanism in the highly defective SnS_2_/HGF sample. However, in the moderately defective SnS_2_/HGF and defect-free SnS/HGF samples, significant contributions from the conversion and alloying mechanisms can be seen from Figs. 7 and 8. Considering the sluggish solid-state diffusion kinetics of Mg^2+^, the Li^+^-driven conversion and alloying reactions play a paramount role in cycle performance of the moderately defective SnS_2_/HGF and defect-free SnS/HGF in MLHBs. As a result, the electrocapacitive contribution due to defective sites of the moderately defective SnS_2_/HGF is not significant. This leads to the observation of a similar cycling performance of the moderately defective SnS_2_/HGF and defect-free SnS/HGF.

## Conclusions

We revealed the role and importance of defects in 2D defective SnS_*x*_ nanosheets in Mg^2+^ and Mg/Li dual-ion storage. A highly defective SnS_2_ encapsulated in 3D holey graphene foams delivered specific capacity as high as ~ 600 mAh g^−1^ at 50 mA g^−1^ with a specific energy density of ~ 330 Wh kg^−1^. The defects in 2D SnS_*x*_ nanosheets provide a large number of active sites for charge storage and creates diffusion channels for ion transport, thus significantly improving the Mg^2+^ storage capacity and transport kinetics. This composite cathode realizes the co-insertion of Mg^2+^ and Li^+^ to enable not only improved cycle stability and capacity, but also decreased amount of electrolyte. The vacancy-like sites in the 2D SnS_*x*_ can absorb considerable stress caused by structural changes upon Mg^2+^ and Li^+^ insertions. The 3D holey graphene foams provide an excellent environment for hosting with charge transport pathways and enhanced electron conductivity.

## Supplementary Information

Below is the link to the electronic supplementary material.

Supplementary file1 (PDF 1531 KB)
