# Supplementary material for: Origin of Excellent Charge Storage Properties of Defective Tin Disulphide in Magnesium/Lithium-Ion Hybrid Batteries
Source: Nanomicro Lett. 2022 Aug 24;14:177. doi: 10.1007/s40820-022-00914-5 (PMC9402882; doi:10.1007/s40820-022-00914-5)
Supplement: Supplementary file 1 — Supplementary file1 (PDF 1531 KB) [file 40820_2022_914_MOESM1_ESM.pdf]

Supporting Information for

Origin of Excellent Charge Storage Properties of Defective Tin Disulfide in Magnesium/Lithium-Ion Hybrid Batteries

Xin Fan[1, 2], Mike Tebyetekerwa[1,3], Yilan Wu[1], Rohit Ranganathan Gaddam[4], and Xiu Song Zhao[1, *]

[1]School of Chemical Engineering, The University of Queensland, St Lucia, Brisbane QLD 4072, Australia

[2]School of Material Science and Technology, North University of China, Shanxi, Taiyuan 030051, P. R. China

[3]Dow Centre for Sustainable Engineering Innovation, School of Chemical Engineering, The University of Queensland, St Lucia, Brisbane QLD 4072, Australia

[4]Department of Chemical Engineering, Indian Institute of Science Education and Research, Bhopal, India

*Corresponding author. E-mail: george.zhao@uq.edu.au (X.S.Z)

Supplementary Figures and Tables

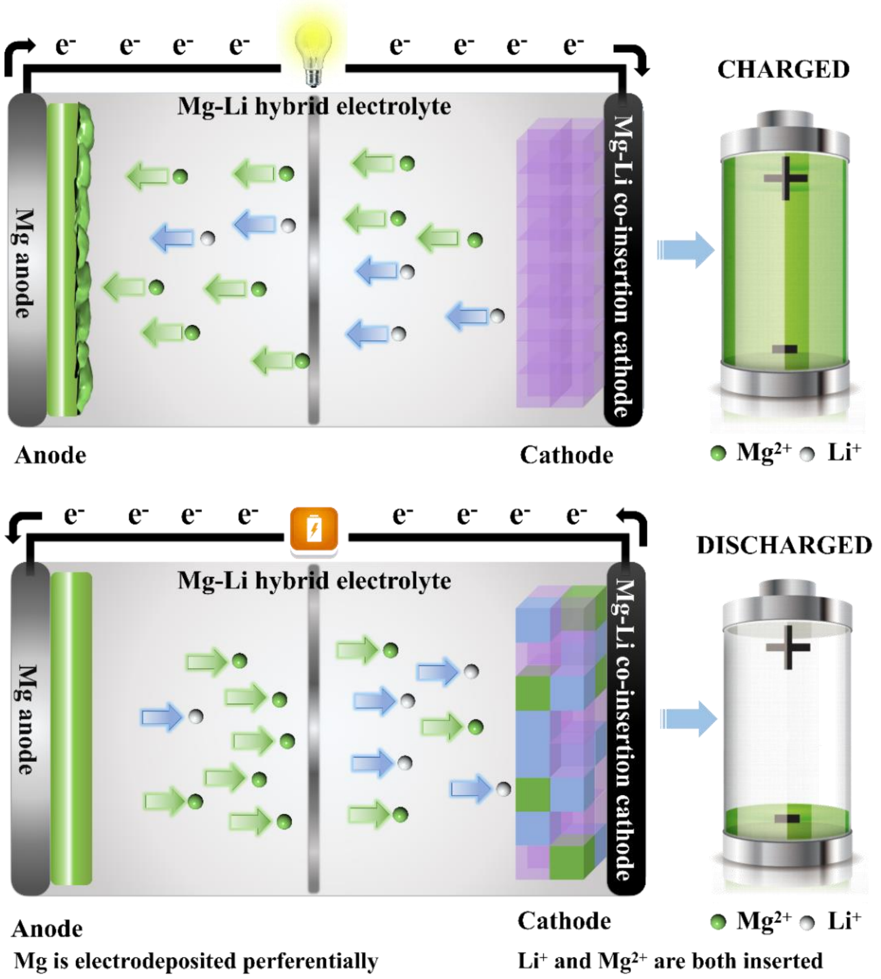

Scheme S1 Schematic illustration of the movement of Mg^{2+} and Li^+ during charge and discharge in a MLHB cell with Mg anode and a Mg^{2+}/Li^+ co-intercalation cathode

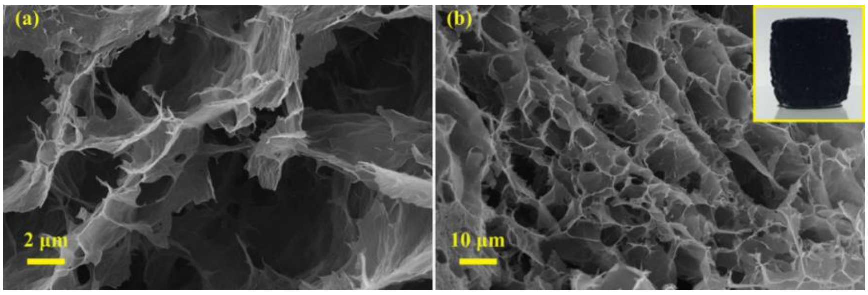

Fig. S1 FESEM images of holey graphene foams (HGF)

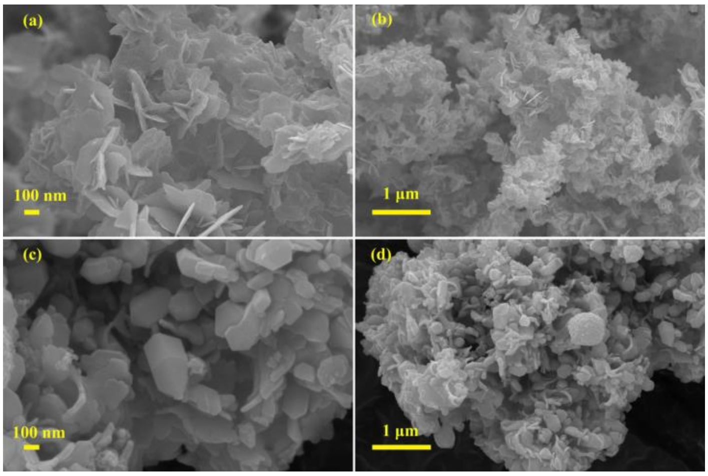

Fig. S2 FESEM images at different magnifications of SnS_2 (**a, b**) and SnS (**c, d**)

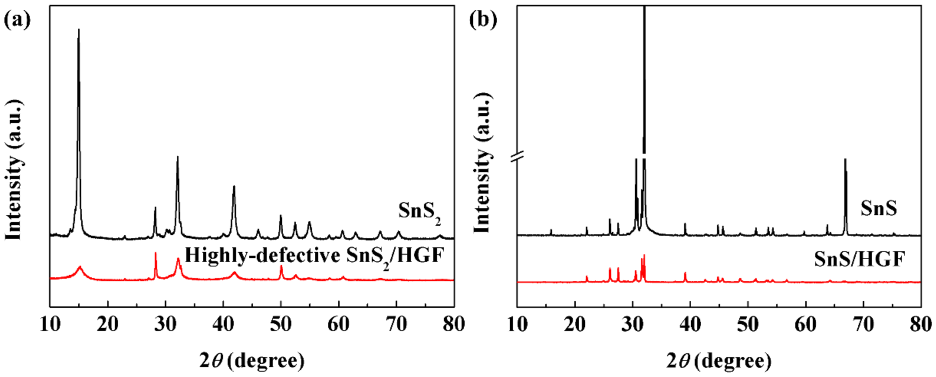

Fig. S3 XRD patterns of (**a**) SnS_2 and highly-defective SnS_2/HGF and of (**b**) SnS and defect-free SnS/HGF (**b**)

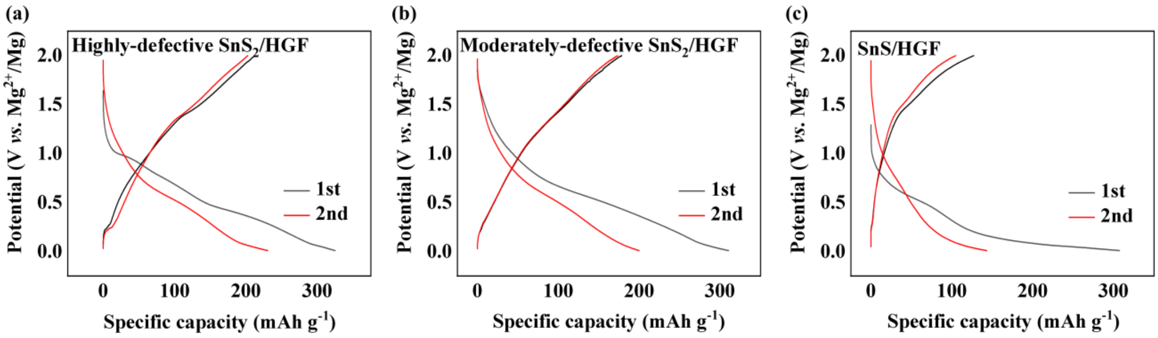

Fig. S4 Galvanostatic discharge and charge profiles of (**a**) highly-defective SnS_2/HGF, (**b**) moderately-defective SnS_2/HGF and (**c**) defect-free SnS/HGF cycled in a MIB at 50 mA g^{-1} in the voltage window between 0.01 and 2.0 V *vs.* Mg^{2+}/Mg

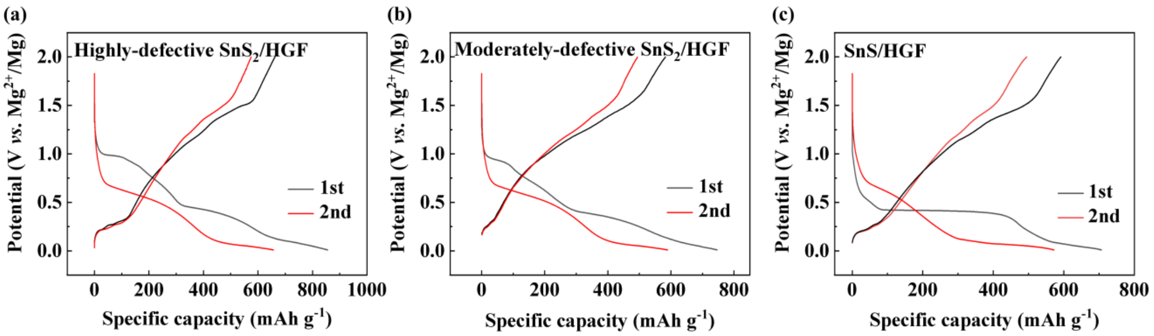

Fig. S5 Galvanostatic discharge and charge profils of (**a**) highly-defective SnS_2/HGF, (**b**) moderately-defective SnS_2/HGF and (**c**) defect-free SnS/HGF cycled in a MLHB at 50 mA g^{-1} in the voltage range 0.01-2.0 V *vs*. Mg^{2+}/Mg

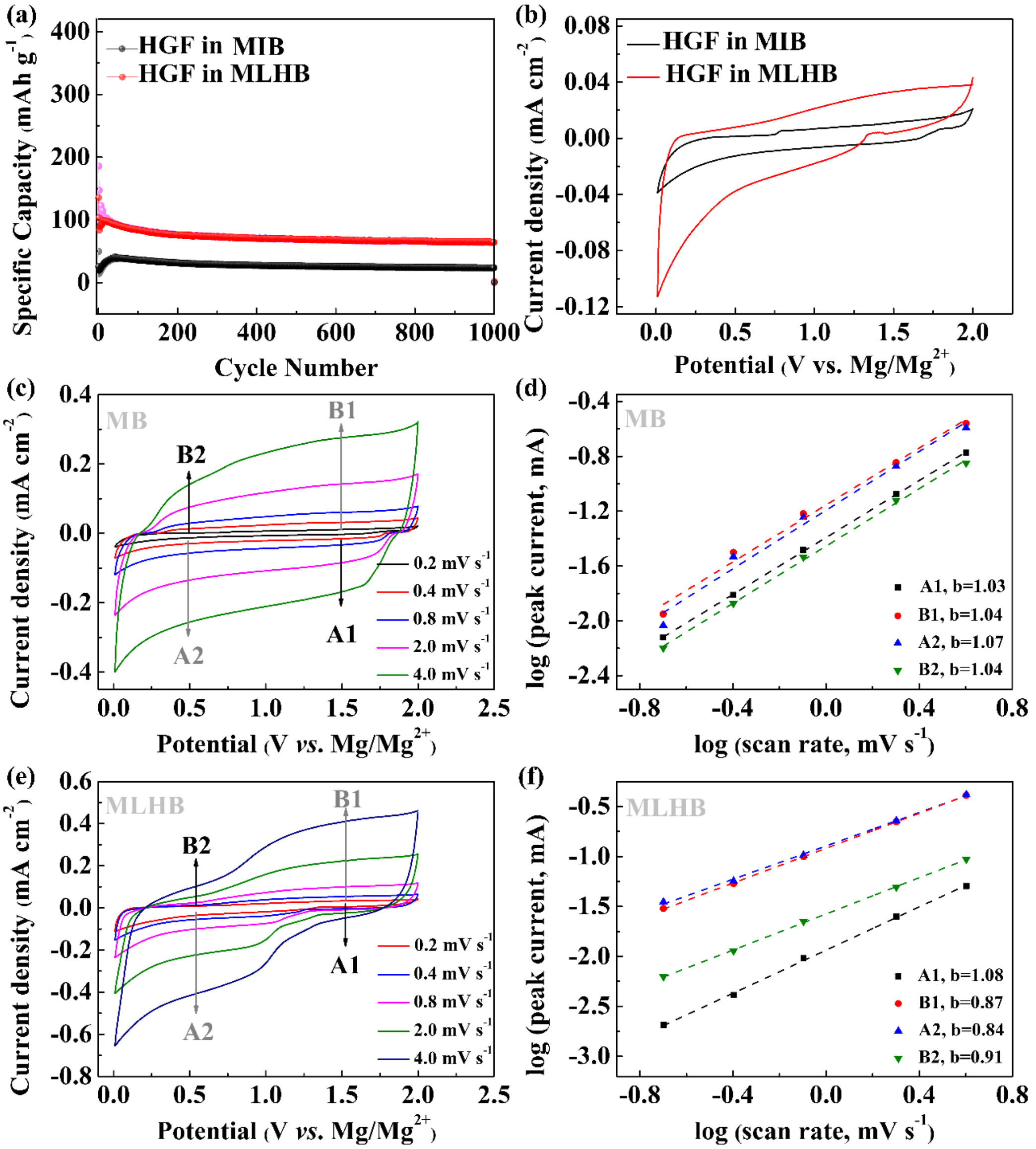

Fig. S6 (**a**) Cycling performance and (**b**) CV curves of HGF in MIBs and MLHBs, showing negligible capacity contribution from HGF. (**c-f**) CV curves at different scan rates to determine *b*-values at different potentials *vs*. Mg^{2+}/Mg in MIBs (c, d) and MLHBs (**e, f**). Constant *b* can be obtained by plotting log $i(V)$ *vs*. log v according to $i(V) = av^b$, where Constant *a* is a constant, indicating a capacity contribution mainly from charge transfer with surface/subsurface atoms

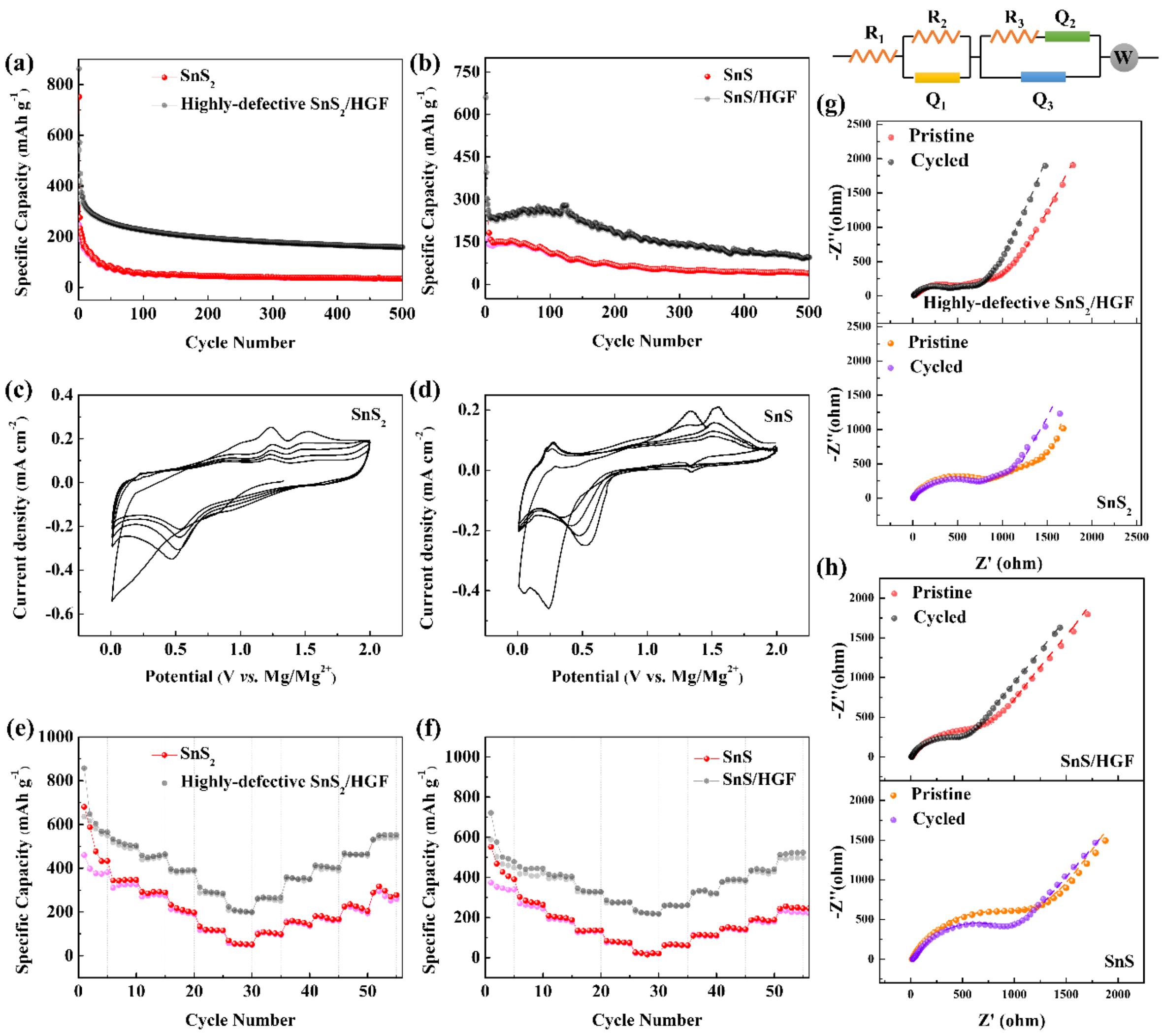

Fig. S7 Comparison of cycling stability between SnS_2 and highly-defective SnS_2/HGF (**a**) and between SnS and SnS/HGF (**b**) at 800 mA g^{-1} in the voltage range between 0.01 and 2.0 V *vs.* Mg^{2+}/Mg. Cyclic voltammograms of SnS_2 (**c**) and SnS (**d**) at the scan rate of 0.2 mV s^{-1}. Comparison of rate performance between SnS_2 and highly-defective SnS_2/HGF (**e**) and between SnS and SnS/HGF (**f**). Comparison of Nyquist plots between SnS_2 and highly-defective SnS_2/HGF (**g**) and between SnS and SnS/HGF (**h**) before and after first cycle at the OCP. The dotted lines were fitted data using the equivalent circuit shown above **g**

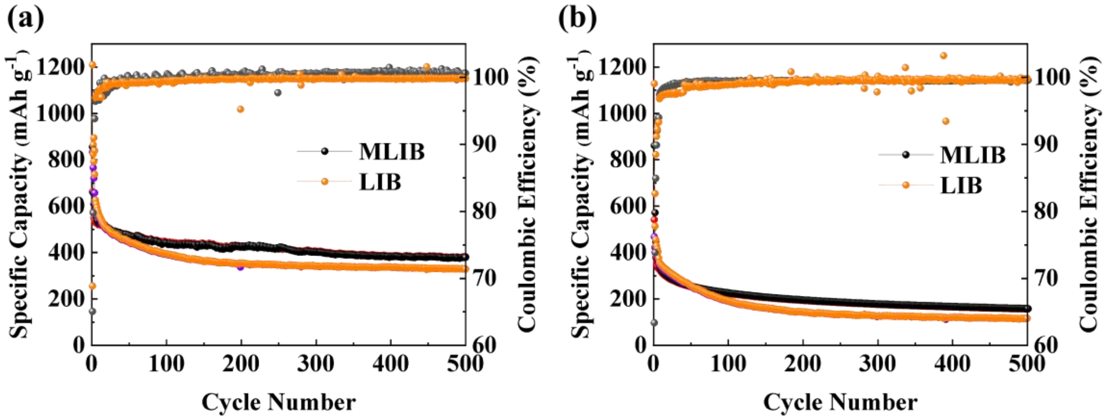

Fig. S8 Cycling performance of highly-defective SnS_2/HGF in LIB in the potential window range between 0.8 and 2.8 V *vs.* Li^+/Li and in MLIB in the potential windows between 0.01 and 2.0 V *vs.* Mg^{2+}/Mg at 50 mA g^{-1} (**a**) and 800 mA g^{-1} (**b**)

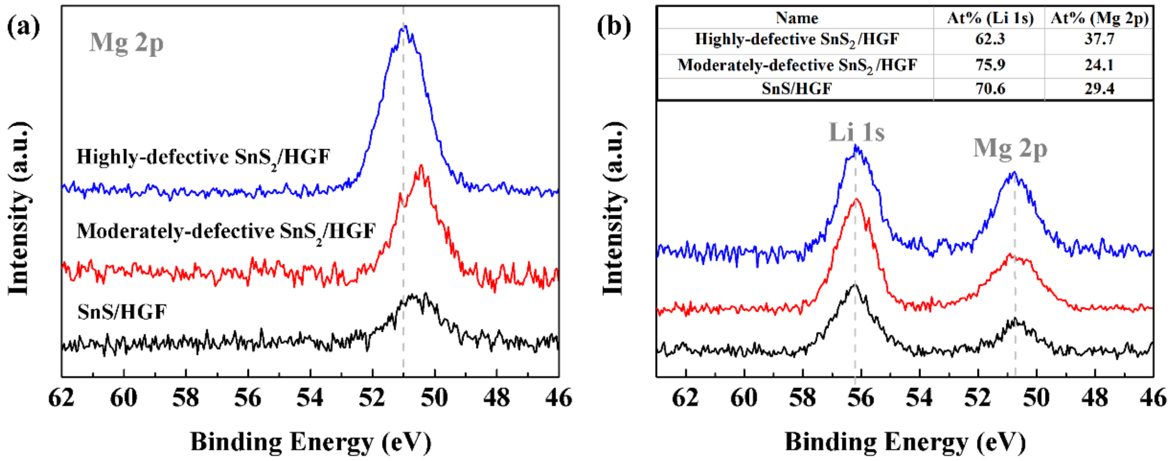

Fig. S9 Mg 2p and Li 1s XPS spectra for fully discharged defective SnS_2/HGF and defect-free SnS/HGF at 0.01 V *vs.* Mg^{2+}/Mg in a MIB cell (**a**) and a MLHB cell (**b**)

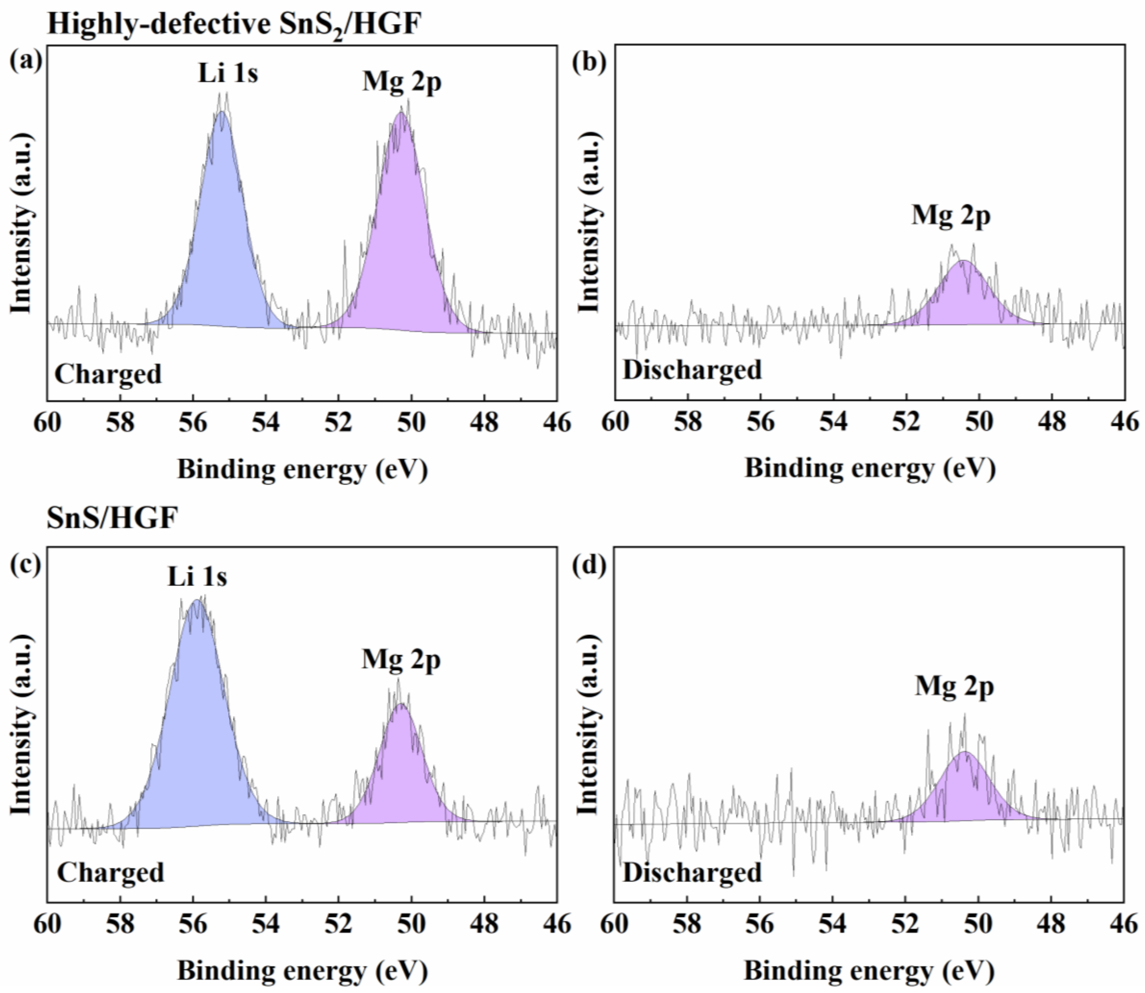

Figure S10 *Ex-situ* XPS spectra represent changes of Mg 2p and Li 1s of the (**a, b**) highly-defective SnS_2/HGF and (**c, d**) SnS/HGF after the first cycle at charged and discharged stages, respectively

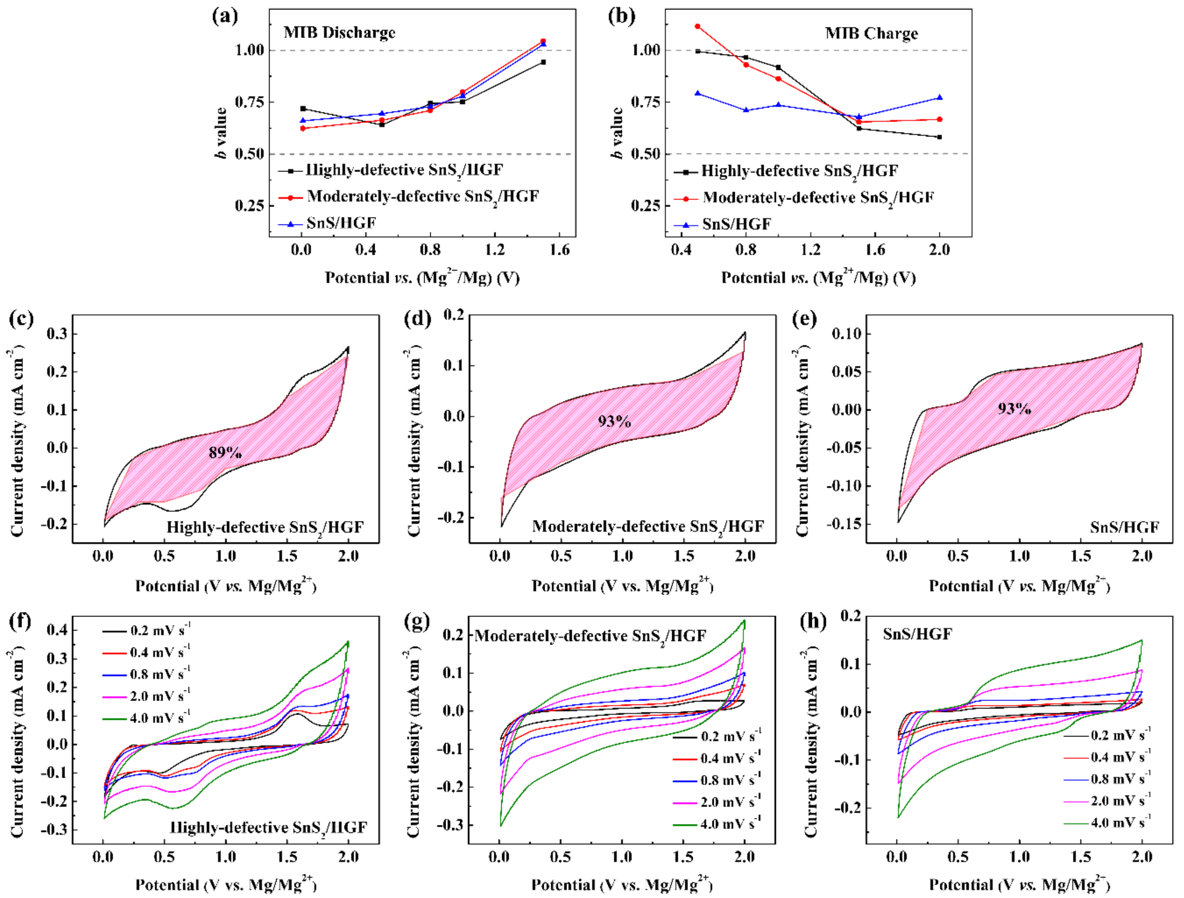

Fig. S11 Kinetics and quantitative analysis of SnS_x/HGF in MIBs. *b*-values of highly-defective SnS_2/HGF, moderately-defective SnS_2/HGF and SnS/HGF at different potentials *vs*. Mg^{2+}/Mg during (**a**) discharging and (**b**) charging processes. CV curves of (**c**) highly-defective SnS_2/HGF, (**d**) moderately-defective SnS_2/HGF and (**e**) SnS/HGF at various scan rates, and the corresponding capacitive charge storage contributions of (**f**) highly-defective SnS_2/HGF, (**g**) moderately-defective SnS_2/HGF and (**h**) SnS/HGF at a scan rate of 2.0 mV s^{-1}

Table S1 Comparison of MLHB cell performance of different electrodes

**Sample**	**Electrolyte**	**Capacity (mAh g^{-1})/current density (mA g^{-1})**	**Refs.**
MoS_2	0.25 M LiCl + 0.25 M APC[a]	301/20	[S1]
TiO_2	1.5 M $LiBH_4$ + 0.5 M $Mg(BH_4)_2$/TGM[b]	140/20	[S2]
$Li_4Ti_5O_{12}$/Graphene	1.5 M $LiBH_4$+0.4 M APC	147.5/20	[S3]
TiS_2	0.4 M LiCl+0.4 M APC	161/20	[S4]
Mo_6S_8	0.4 M LiCl+1 M APC	126/20	[S5]
$MoSe_2$/C	0.5 M LiCl+0.2 M APC	204/50	[S6]
VO_2	1 M LiCl+0.25 M APC	210.6/50	[S7]
VS_2-GO	1 M APC-LiC	235/90	[S8]
d-$Ti_3C_2T_x$/CNT	0.4 M LiCl+0.5 M APC	105/10	[S9]

$TiNb_2O_7$	1.25 M LiCl + 0.4 M APC	240/7.75	[S10]
Cu_2Se	1 M LiCl + 0.4 M APC	239.7/26	[S11]
$Li_3V_2(PO_4)_3$	1 M LiCl + 0.4 M APC	147.8/50	[S12]
$Li_4Mn_5O_{12}$	1 M LiCl + 0.25 M APC	155/16.3	[S13]
FeS_2	1.5 M LiBH4 + 0.1 M Mg(BH4)2 (DGM[c])	600/45	[S14]
FeS/CNF	0.4 M LiCl + 0.4 M APC	463/70	[S15]
Cu_9S_5-AEHPA[d]	1 M LiTFSI + 0.2 M Mg(HMDS)2[e]-AlCl3-MgCl2 (DGM)	280/50	[S16]
LiV_3O_8@GO	1 M LiCl + APC	245.9/50	[S17]
Cu_2Se/rGO	1 M LiCl + 0.4 M APC	243/26	[S18]
$TiNb_2O_7$	1.5 M LiCl + 0.4 M APC	225/7.75	[S19]
Cu_2S@C	1 M LiCl + 0.4 M APC	393.2/16.84	[S20]
Ni-doped MnO_2/CNT	1 M LiCl + 0.4 M APC	175/20	[S21]
$(NiMnCo)_3O_4$	1 M LiCl + 0.4 M APC	550/50	[S22]
$Na_2C_6O_6$	1 M LiCl + 0.25 M APC	350/50	[S23]
SnS_2/HGF	0.25 M LiCl + 0.25 M APC	600/50	This work
SnS/HGF	0.25 M LiCl + 0.25 M APC	520/50	

* [a]APC: All-phenyl complex. [b]TGM: Tetraglyme. [c]DGM: Diglyme. [d]AEHPA: Amino-ended hyperbranched polyamide. [e]$Mg(HMDS)_2$: Bishexamethyldisilazide magnesium.

Supplementary References

[S1] X. Fan, R.R. Gaddam, N.A. Kumar, X.S. Zhao, A hybrid Mg^{2+}/Li^+ battery based on interlayer-expanded MoS_2/graphene cathode. Adv. Energy Mater. **7**(19), 1700317 (2017). https://doi.org/10.1002/aenm.201700317

[S2] S. Su, Z. Huang, Y. NuLi, F. Tuerxun, J. Yang et al., A novel rechargeable battery with a magnesium anode, a titanium dioxide cathode, and a magnesium borohydride/tetraglyme electrolyte. Chem. Commun. **51**(13), 2641-2644 (2015). https://doi.org/10.1039/C4CC08774G

[S3] Q. Miao, Y. NuLi, N. Wang, J. Yang, J. Wang et al, Effect of Mg^{2+}/Li^+ mixed electrolytes on a rechargeable hybrid battery with $Li_4Ti_5O_{12}$ cathode and Mg anode. RSC Adv. **6**(4), 3231-3234 (2016). https://doi.org/10.1039/C5RA25827H

[S4] T. Gao, F. Han, Y. Zhu, L. Suo, C. Luo et al., Hybrid Mg^{2+}/Li^+ battery with long cycle life and high rate capability. Adv. Energy Mater. **5**(5), 1401507 (2015). https://doi.org/10.1002/aenm.201401507

[S5] Y. Cheng, Y. Shao, J.G. Zhang, V.L. Sprenkle, J. Liu et al., High performance batteries based on hybrid magnesium and lithium chemistry. Chem. Commun. **50**, 9644-9646 (2014). https://doi.org/10.1039/C4CC03620D

[S6] J.J. Fan, S.Y. Shen, Y. Chen, L.N. Wu, J. Peng et al., A rechargeable Mg^{2+}/Li^{+} hybrid battery based on sheet-like $MoSe_2$/C nanocomposites cathode. Electrochem. Commun. **90**, 16-20 (2018). https://doi.org/10.1016/j.elecom.2018.03.004

[S7] C. Pei, F. Xiong, J. Sheng, Y. Yin, S. Tan et al., VO_2 nanoflakes as the cathode material of hybrid magnesium-lithium-ion batteries with high energy density. ACS Appl. Mater. Interfaces **9**(20), 17060-17066 (2017). https://doi.org/10.1021/acsami.7b02480

[S8] R. Sun, C. Pei, J. Sheng, D. Wang, L. Wu et al., High-rate and long-life VS_2 cathodes for hybrid magnesium-based battery. Energy Storage Mater. **12**, 61-68 (2018). https://doi.org/10.1016/j.ensm.2017.11.012

[S9] A. Byeon, M.Q. Zhao, C.E. Ren, J. Halim, S. Kota et al., Two-dimensional titanium carbide MXene as a cathode material for hybrid magnesium/lithium-ion batteries. ACS Appl. Mater. Interfaces **9**(5), 4296-4300 (2017). https://doi.org/10.1021/acsami.6b04198

[S10] S. Maletti, O. Janson, A. Herzog-Arbeitman, I.G.G. Martinez, R. Buckan et al., Operation mechanism in hybrid Mg-Li batteries with $TiNb_2O_7$ allowing stable high-rate cycling ACS Appl. Mater. Interfaces **13**(5), 6309-6321 (2021). https://doi.org/10.1021/acsami.0c20905

[S11] H.C. Yuan, N. Wang, Y.N. NuLi, J. Yang, J.L. Wang, Hybrid Mg^{2+}/Li^{+} batteries with Cu_2Se cathode based on displacement reaction. Electrochim. Acta **261**, 503-512 (2018). https://doi.org/10.1016/j.electacta.2017.12.169

[S12] M. Rashad, H. Zhang, X. Li, H. Zhang, J. Mater, Fast kinetics of Mg^{2+}/Li^{+} hybrid ions in a polyanion $Li_3V_2(PO_4)_3$ cathode in a wide temperature range. J. Mater. Chem. A **7**(16), 9968-9976 (2019). https://doi.org/10.1039/C9TA00502A

[S13] Y. Cen, Y. Liu, Y. Zhou, L. Tang, P. Jiang et al., Spinel $Li_4Mn_5O_{12}$ as 2.0 V insertion materials for Mg-based hybrid ion batteries. ChemElectroChem **7**(5), 1115-1124 (2020). https://doi.org/10.1002/celc.201902105

[S14] Y. Zhang, J. Xie, Y. Han, C. Li, Dual-salt Mg-based Batteries with conversion cathodes. Adv. Funct. Mater. **25**(47), 7300-7308 (2015). https://doi.org/10.1002/adfm.201503639

[S15] X. Chen, S. Wang, H. Wang, High performance hybrid Mg-Li ion batteries with conversion cathodes for low cost energy storage. Electrochim. Acta **265**, 175-183 (2018). https://doi.org/10.1016/j.electacta.2018.01.148

[S16] T. Li, A. Qin, H. Wang, M. Wu, Y. Zhang et al., A high-performance hybrid Mg^{2+}/Li^{+} battery based on hierarchical copper sulfide microflowers conversion cathode. Electrochim. Acta **263**, 168-175 (2018). https://doi.org/10.1016/j.electacta.2018.01.067

[S17] M. Li, C. Pei, F. Xiong, S. Tan, Y. Yin et al., A high energy density hybrid magnesium-lithium ion battery based on LiV_3O_8@GO cathode. Electrochim. Acta **320**, 134556 (2019). https://doi.org/10.1016/j.electacta.2019.134556

[S18] H. Yuan, N. Wang, Y. NuLi, J. Yang, J. Wang, Hybrid Mg^{2+}/Li^{+} batteries with Cu_2Se cathode based on displacement reaction. Electrochim. Acta **261**, 503-512 (2018). https://doi.org/10.1016/j.electacta.2017.12.169

[S19] S. Maletti, A. Herzog-Arbeitman, S. Oswald, A. Senyshyn, L. Giebeler et al., $TiNb_2O_7$ and VNb_9O_{25} of ReO_3 type in hybrid Mg–Li batteries: electrochemical and interfacial

insights. J. Phys. Chem. C **124**(46), 25239-25248 (2020). https://doi.org/10.1021/acs.jpcc.0c07373

[S20] W. Wang, Y. Yang, Y. NuLi, J. Zhou, J. Yang et al., Metal organic framework (MOF)-derived carbon-encapsulated cuprous sulfide cathode based on displacement reaction for hybrid Mg^{2+}/Li^{+} batteries. J. Power Sources **445**, 227325 (2020). https://doi.org/10.1016/j.jpowsour.2019.227325

[S21] M. Asif, M. Rashad, Z. Ali, H. Qiu, W. Li et al., Ni-doped MnO_2/CNT nanoarchitectures as a cathode material for ultra-long life magnesium/lithium hybrid ion batteries. Mater. Today Energy **10**, 108-117 (2018). https://doi.org/10.1016/j.mtener.2018.08.010

[S22] M. Asif, M. Rashad, Z. Ali, I. Ahmed, Synthesis of ternary metal oxides as positive electrodes for Mg-Li hybrid ion batteries. Nanoscale **12**(2), 924-932 (2020). https://doi.org/10.1039/C9NR08758C

[S23] J. Tian, D. Cao, X. Zhou, J. Hu, M. Huang et al., High-capacity Mg-organic batteries based on nanostructured rhodizonate salts activated by Mg-Li dual-salt electrolyte. ACS Nano **12**(4), 3424-3435 (2018). https://doi.org/10.1021/acsnano.7b09177
